# Supplementary material for: The lysine‐specific methyltransferase KMT2C/MLL3 regulates DNA repair components in cancer
Source: EMBO Rep. 2019 Jan 21;20(3):e46821. doi: 10.15252/embr.201846821 (PMC6399616; doi:10.15252/embr.201846821)
Supplement: Supplementary file 1 — Appendix [file EMBR-20-e46821-s001.docx]

**Appendix**

**Appendix Table 1……………………………………………………………………………2**

**Appendix Table 2……………………………………………………………………………3**

**Appendix Table 3……………………………………………………………………………6**

**Appendix Table 4…………………………………………………………………………..14**

**Appendix Table 5…………………………………………………………………………..15**

**Appendix Table 6……………………………………………………………………..……16**

**Appendix Table 7……………………………..……………………………………………17**

**Appendix Figure 1…………………………..……………………………………………..18**

**Appendix Figure 2…………………………..……………………………………………..19**

**Appendix Figure 3………………………………………………………………..………..20**

**Appendix Figure 4………………………………………………………………..………..21**

**Appendix Figure 5………………………………………………………………..………..22**

**Appendix Table S1.** Clinicopathological features of the BC patients

| **Variable** | **No. of patients N=138** |
| --- | --- |
| **Disease**  Non-muscle-invasive bladder cancer (NMIBC)  Muscle-invasive bladder cancer (MIBC) | **94** (68.1%)  **44** (31.9%) |
| **Tumor stage**  pTa  pT1  pT2  pT3  pT4 | **50** (36.2%)  **44** (31.9%)  **20** (14.5%)  **12** (8.7%)  **12** (8.7%) |
| **Grade (WHO 2004)**  Low  High | **65** (47.1%)  **73** (52.9%) |
| **Grade (WHO 1973)**  1  2  3 | **16** (11.6%)  **55** (39.9%)  **67** (48.6%) |
| **Gender**  Male  Female | **118** (85.5%)  **20** (14.5%) |
| **EORTC-risk stratification of NMIBC**  Low risk  Intermediate risk  High risk | **17** (18.1%)  **25** (26.6%)  **52** (55.3%) |
| **Variable** | **Median (Range)** |
| **Age**  (age unknown for 18 patients) | **71.0** (45 – 88) |

**Appendix Table S2.** Patient list

| **Patient** | **Grade**  **(WHO 1973)** | **Grade**  **(WHO 2004)** | **Tumor Stage** | **Age**  **(years)** | **Gender** |
| --- | --- | --- | --- | --- | --- |
| UCC1 | 3 | High | T2 | 67 | F |
| UCC2 | 3 | High | T2 | 76 | M |
| UCC3 | 2 | Low | Ta | 84 | F |
| UCC4 | 3 | High | T1 | 76 | M |
| UCC5 | 3 | High | T3 | 66 | M |
| UCC6 | 3 | High | T3 | 79 | M |
| UCC7 | 2 | Low | Ta | 68 | M |
| UCC8 | 1 | Low | Ta | 62 | M |
| UCC9 | 3 | High | T3 | 54 | M |
| UCC11 | 1 | Low | Ta | 72 | M |
| UCC12 | 2 | Low | T1 | 65 | M |
| UCC13 | 1 | Low | Ta | 78 | M |
| UCC14 | 3 | High | T2 | 45 | M |
| UCC15 | 2 | Low | Ta | 82 | M |
| UCC16 | 3 | High | T3 | 74 | M |
| UCC17 | 3 | High | T1 | 88 | M |
| UCC18 | 1 | Low | Ta | 79 | M |
| UCC19 | 1 | Low | Ta | 71 | M |
| UCC20 | 3 | High | T4 | Unknown | M |
| UCC22 | 3 | High | T2 | 80 | F |
| UCC23 | 3 | High | T3 | 88 | M |
| UCC24 | 3 | High | T1 | 78 | M |
| UCC25 | 3 | High | T3 | 71 | M |
| UCC26 | 2 | Low | T1 | 76 | M |
| UCC27 | 2 | Low | T2 | 63 | M |
| UCC28 | 2 | Low | T1 | 61 | M |
| UCC29 | 1 | Low | Ta | 76 | M |
| UCC30 | 3 | High | T4 | 72 | F |
| UCC31 | 3 | High | T1 | 71 | M |
| UCC32 | 2 | High | T1 | 76 | M |
| UCC33 | 2 | Low | Ta | 73 | M |
| UCC34 | 2 | Low | Ta | 85 | M |
| UCC35 | 3 | High | T2 | Unknown | M |
| UCC36 | 1 | Low | Ta | 58 | M |
| UCC37 | 3 | High | T1 | 78 | M |
| UCC38 | 3 | High | T3 | 72 | M |
| UCC39 | 2 | Low | T1 | 62 | M |
| UCC40 | 2 | Low | Ta | 58 | M |
| UCC41 | 3 | High | T2 | 85 | M |
| UCC43 | 3 | High | T2 | 64 | M |
| UCC44 | 2 | Low | Ta | 59 | M |
| UCC45 | 3 | High | T3 | 69 | M |
| UCC46 | 2 | Low | T1 | Unknown | M |
| UCC47 | 2 | Low | Ta | 63 | M |
| UCC48 | 3 | High | T3 | 81 | M |
| UCC49 | 1 | Low | T1 | 52 | F |
| UCC50 | 3 | High | T4 | 79 | M |
| UCC51 | 3 | High | T4 | 62 | M |
| UCC52 | 2 | High | Ta | 69 | M |
| UCC53 | 3 | High | T3 | 80 | M |
| UCC54 | 2 | High | T1 | 69 | M |
| UCC55 | 2 | Low | T1 | Unknown | M |
| UCC56 | 1 | Low | Ta | 50 | M |
| UCC57 | 2 | Low | Ta | Unknown | M |
| UCC58 | 3 | High | T4 | 71 | M |
| UCC59 | 2 | Low | Ta | 85 | M |
| UCC60 | 2 | Low | Ta | 78 | F |
| UCC61 | 1 | Low | Ta | 63 | M |
| UCC65 | 3 | High | T1 | 66 | M |
| UCC67 | 3 | High | T4 | 74 | M |
| UCC68 | 2 | High | T1 | 56 | M |
| UCC69 | 3 | High | T4 | 60 | M |
| UCC70 | 2 | Low | Ta | 57 | M |
| UCC71 | 2 | Low | T1 | 54 | M |
| UCC73 | 3 | High | T3 | 72 | M |
| UCC75 | 2 | Low | Ta | Unknown | M |
| UCC76 | 1 | Low | Ta | Unknown | M |
| UCC77 | 3 | High | T1 | 73 | M |
| UCC78 | 2 | Low | Ta | 64 | M |
| UCC79 | 3 | High | T1 | Unknown | M |
| UCC80 | 3 | High | T2 | 74 | M |
| UCC81 | 3 | High | T1 | 72 | M |
| UCC82 | 3 | High | T1 | 81 | M |
| UCC84 | 1 | Low | Ta | 63 | M |
| UCC85 | 2 | Low | Ta | 85 | M |
| UCC86 | 3 | High | Ta | 68 | M |
| UCC87 | 1 | Low | T1 | 83 | F |
| UCC89 | 2 | Low | T1 | 86 | M |
| UCC90 | 2 | Low | T1 | 73 | M |
| UCC91 | 3 | High | T2 | 75 | M |
| UCC92 | 3 | High | T1 | 60 | M |
| UCC93 | 3 | High | T1 | 62 | M |
| UCC95 | 3 | High | T1 | 58 | F |
| UCC96 | 2 | Low | T2 | 75 | M |
| UCC98 | 3 | High | T2 | 65 | M |
| UCC100 | 3 | High | T1 | 54 | F |
| UCC102 | 3 | High | Ta | 56 | M |
| UCC107 | 2 | Low | Ta | 61 | F |
| UCC109 | 3 | High | T1 | Unknown | M |
| UCC111 | 2 | Low | Ta | Unknown | M |
| UCC112 | 2 | High | T1 | 71 | M |
| UCC113 | 2 | Low | T1 | 77 | M |
| UCC114 | 2 | Low | Ta | 48 | M |
| UCC115 | 2 | Low | Ta | Unknown | M |
| UCC116 | 2 | Low | Ta | 87 | M |
| UCC117 | 2 | Low | Ta | 54 | F |
| UCC118 | 2 | Low | Ta | 61 | M |
| UCC119 | 2 | Low | T1 | Unknown | M |
| UCC126 | 1 | Low | Ta | 78 | M |
| UCC127 | 3 | High | T2 | 85 | M |
| UCC128 | 3 | High | T1 | Unknown | F |
| UCC129 | 2 | High | T1 | Unknown | M |
| UCC130 | 3 | High | Ta | 50 | M |
| UCC131 | 2 | Low | T1 | 62 | M |
| UCC132 | 2 | Low | Ta | 70 | M |
| UCC133 | 2 | High | T1 | 85 | M |
| UCC134 | 3 | High | T1 | 60 | F |
| UCC136 | 2 | Low | Ta | 85 | M |
| UCC137 | 1 | Low | Ta | 64 | F |
| UCC138 | 2 | Low | T1 | 76 | M |
| UCC139 | 3 | High | T1 | 81 | F |
| UCC140 | 2 | Low | Ta | 68 | F |
| UCC141 | 3 | High | T2 | 75 | M |
| UCC142 | 3 | High | T1 | 76 | M |
| UCC143 | 3 | High | T2 | 59 | M |
| UCC144 | 3 | High | T2 | 72 | F |
| UCC145 | 3 | High | T4 | 75 | M |
| UCC146 | 3 | High | T2 | 88 | M |
| UCC147 | 3 | High | T4 | 59 | M |
| UCC148 | 2 | Low | Ta | Unknown | M |
| UCC150 | 3 | High | T4 | 58 | M |
| UCC151 | 3 | High | Ta | 60 | M |
| UCC153 | 3 | High | Ta | 68 | F |
| UCC154 | 2 | Low | T1 | 69 | M |
| UCC156 | 3 | High | T4 | 73 | M |
| UCC157 | 2 | Low | Ta | 79 | M |
| UCC159 | 3 | High | T1 | 74 | M |
| UCC160 | 2 | Low | T4 | 67 | M |
| UCC161 | 2 | Low | Ta | 63 | F |
| UCC162 | 3 | High | Ta | 56 | M |
| UCC163 | 2 | Low | T1 | Unknown | M |
| UCC164 | 3 | High | T2 | 51 | F |
| UCC166 | 2 | High | T2 | 59 | M |
| UCC167 | 1 | Low | Ta | 61 | M |
| UCC168 | 2 | Low | Ta | 64 | M |
| UCC169 | 3 | High | T3 | Unknown | M |
| UCC170 | 2 | Low | T1 | Unknown | M |
| UCC171 | 3 | High | T2 | 83 | M |

| **Appendix Table S3**. Raw tumor measurements of xenografts tumors  **HTB9 Scr** | | | | | | | | | | | | | | | | | |
| --- | --- | --- | --- | --- | --- | --- | --- | --- | --- | --- | --- | --- | --- | --- | --- | --- | --- |
| **Olaparib** | | | | | | | | | | | | | | | | | |
|  | *Width* | | | | | | | | *Length* | | | | | | | | *Net Weight* |
|  | **Day** |  |  |  |  |  |  |  | **Day** |  |  |  |  |  |  |  | **Day** |
| **Tumor** | *D1* | *D4* | *D7* | *D10* | *D13* | *D16* | *D19* | *D22* | *D1* | *D4* | *D7* | *D10* | *D13* | *D16* | *D19* | *D22* | *D22* |
| *1* | 0.1 | 0.3 | 0.4 | 0.5 | 0.5 | 0.3 | 0.4 | 0.4 | 0.3 | 0.6 | 0.5 | 0.5 | 0.5 | 0.5 | 0.6 | 0.6 | 0.150 |
| *2* | 0.3 | 0.6 | 0.6 | 0.6 | 0.6 | 0.7 | 0.8 | 0.9 | 0.3 | 0.6 | 0.6 | 0.7 | 0.7 | 0.8 | 0.9 | 0.9 | 0.592 |
| *3* | 0.2 | 0.3 | 0.4 | 0.5 | 0.5 | 0.7 | 0.6 | 0.7 | 0.2 | 0.3 | 0.4 | 0.6 | 0.6 | 0.9 | 0.7 | 0.8 | 0.236 |
| *4* | 0.3 | 0.4 | 0.5 | 0.6 | 0.8 | 0.9 | 0.9 | 0.9 | 0.5 | 0.9 | 1.1 | 1.2 | 1.3 | 1.3 | 1.3 | 1.4 | 0.740 |
| *5* | 0.3 | 0.4 | 0.5 | 0.6 | 0.6 | 0.8 | 0.8 | 0.8 | 0.4 | 0.6 | 0.7 | 1.1 | 1.2 | 1.3 | 1.4 | 1.5 | 0.743 |
| *6* | 0.3 | 0.4 | 0.5 | 0.6 | 0.6 | 0.9 | 0.9 | 0.9 | 0.3 | 0.4 | 0.5 | 0.7 | 0.7 | 0.9 | 0.9 | 1.1 | 0.587 |
| *7* | 0.1 | 0.3 | 0.3 | 0.4 | 0.4 | 0.5 | 0.6 | 0.6 | 0.2 | 0.3 | 0.3 | 0.5 | 0.5 | 0.7 | 0.7 | 0.8 | 0.226 |
| **Mean** | 0.229 | 0.386 | 0.457 | 0.543 | 0.571 | 0.686 | 0.714 | 0.743 | 0.314 | 0.529 | 0.586 | 0.757 | 0.786 | 0.914 | 0.929 | 1.014 | 0.468 |
| **SD** | 0.095 | 0.107 | 0.098 | 0.079 | 0.125 | 0.219 | 0.186 | 0.190 | 0.107 | 0.214 | 0.261 | 0.282 | 0.329 | 0.297 | 0.309 | 0.334 | 0.256 |
| **SE** | 0.036 | 0.040 | 0.037 | 0.030 | 0.047 | 0.083 | 0.070 | 0.072 | 0.040 | 0.081 | 0.099 | 0.107 | 0.124 | 0.112 | 0.117 | 0.126 | 0.097 |
| **Vehicle** | | | | | | | | | | | | | | | | | |
|  | *Width* | | | | | | | | *Length* | | | | | | | | *Net Weight* |
|  | **Day** |  |  |  |  |  |  |  | **Day** |  |  |  |  |  |  |  | **Day** |
| **Tumor** | *D1* | *D4* | *D7* | *D10* | *D13* | *D16* | *D19* | *D22* | *D1* | *D4* | *D7* | *D10* | *D13* | *D16* | *D19* | *D22* | *D22* |
| *1* | 0.2 | 0.4 | 0.8 | 0.9 | 1.0 | 1.2 | 1.2 | 1.2 | 0.3 | 0.6 | 0.8 | 0.9 | 1.1 | 1.2 | 1.2 | 1.2 | 0.843 |
| *2* | 0.4 | 0.7 | 0.8 | 1.0 | 1.2 | 1.4 | 1.4 | 1.4 | 0.4 | 0.8 | 0.8 | 1.0 | 1.2 | 1.4 | 1.5 | 1.6 | 1.391 |
| *3* | 0.3 | 0.5 | 0.7 | 0.8 | 1.0 | 1.1 | 1.1 | 1.1 | 0.3 | 0.5 | 0.7 | 0.9 | 1.0 | 1.1 | 1.2 | 1.2 | 0.539 |
| *4* | 0.2 | 0.4 | 0.5 | 0.7 | 0.7 | 0.9 | 0.9 | 0.9 | 0.3 | 0.6 | 0.6 | 0.8 | 0.8 | 0.9 | 0.9 | 0.9 | 0.258 |
| *5* | 0.3 | 0.6 | 0.8 | 1.0 | 1.0 | 1.1 | 1.1 | 1.1 | 0.3 | 0.6 | 0.8 | 1.2 | 1.2 | 1.4 | 1.4 | 1.4 | 0.838 |
| *6* | 0.3 | 0.6 | 0.8 | 1.1 | 1.2 | 1.2 | 1.3 | 1.3 | 0.4 | 0.7 | 1.0 | 1.2 | 1.3 | 1.4 | 1.4 | 1.4 | 0.879 |
| **Mean** | 0.283 | 0.533 | 0.733 | 0.917 | 1.017 | 1.150 | 1.167 | 1.167 | 0.333 | 0.633 | 0.783 | 1.000 | 1.100 | 1.233 | 1.267 | 1.283 | 0.791 |
| **SD** | 0.075 | 0.121 | 0.121 | 0.147 | 0.183 | 0.164 | 0.175 | 0.175 | 0.052 | 0.103 | 0.133 | 0.167 | 0.179 | 0.207 | 0.216 | 0.240 | 0.380 |
| **SE** | 0.031 | 0.049 | 0.049 | 0.060 | 0.075 | 0.067 | 0.071 | 0.071 | 0.021 | 0.042 | 0.054 | 0.068 | 0.073 | 0.084 | 0.088 | 0.098 | 0.155 |

| **HTB9 KD1** | | | | | | | | | | | | | | | | | |
| --- | --- | --- | --- | --- | --- | --- | --- | --- | --- | --- | --- | --- | --- | --- | --- | --- | --- |
| **Olaparib** | | | | | | | | | | | | | | | | | |
|  | *Width* | | | | | | | | *Length* | | | | | | | | *Net Weight* |
|  | **Day** |  |  |  |  |  |  |  | **Day** |  |  |  |  |  |  |  | **Day** |
| **Tumor** | *D1* | *D4* | *D7* | *D10* | *D13* | *D16* | *D19* | *D22* | *D1* | *D4* | *D7* | *D10* | *D13* | *D16* | *D19* | *D22* | *D22* |
| *1* | 0.2 | 0.2 | 0.3 | 0.4 | 0.5 | 0.5 | 0.4 | 0.4 | 0.2 | 0.2 | 0.3 | 0.4 | 0.5 | 0.5 | 0.5 | 0.5 | 0.145 |
| *2* | 0.2 | 0.2 | 0.3 | 0.4 | 0.5 | 0.5 | 0.5 | 0.5 | 0.2 | 0.2 | 0.3 | 0.4 | 0.5 | 0.6 | 0.6 | 0.6 | 0.108 |
| *3* | 0.1 | 0.1 | 0.2 | 0.3 | 0.3 | 0.4 | 0.3 | 0.3 | 0.1 | 0.1 | 0.2 | 0.3 | 0.3 | 0.4 | 0.3 | 0.3 | 0.034 |
| *4* | 0.2 | 0.2 | 0.2 | 0.3 | 0.3 | 0.3 | 0.2 | 0.2 | 0.2 | 0.2 | 0.2 | 0.3 | 0.3 | 0.3 | 0.2 | 0.2 | 0.019 |
| *5* | 0.3 | 0.2 | 0.2 | 0.2 | 0.3 | 0.3 | 0.2 | 0.2 | 0.3 | 0.3 | 0.3 | 0.3 | 0.3 | 0.3 | 0.3 | 0.3 | 0.031 |
| *6* | 0.3 | 0.1 | 0.2 | 0.3 | 0.3 | 0.3 | 0.2 | 0.2 | 0.3 | 0.2 | 0.2 | 0.3 | 0.3 | 0.4 | 0.2 | 0.2 | 0.019 |
| *7* | 0.2 | 0.2 | 0.3 | 0.3 | 0.4 | 0.4 | 0.4 | 0.4 | 0.2 | 0.3 | 0.4 | 0.5 | 0.6 | 0.6 | 0.5 | 0.5 | 0.149 |
| **Mean** | 0.214 | 0.171 | 0.243 | 0.314 | 0.371 | 0.386 | 0.314 | 0.314 | 0.214 | 0.214 | 0.271 | 0.357 | 0.400 | 0.443 | 0.371 | 0.371 | 0.072 |
| **SD** | 0.069 | 0.049 | 0.053 | 0.069 | 0.095 | 0.090 | 0.121 | 0.121 | 0.069 | 0.069 | 0.076 | 0.079 | 0.129 | 0.127 | 0.160 | 0.160 | 0.060 |
| **SE** | 0.026 | 0.018 | 0.020 | 0.026 | 0.036 | 0.034 | 0.046 | 0.046 | 0.026 | 0.026 | 0.029 | 0.030 | 0.049 | 0.048 | 0.061 | 0.061 | 0.023 |
| **Vehicle** | | | | | | | | | | | | | | | | | |
|  | *Width* | | | | | | | | *Length* | | | | | | | | *Net Weight* |
|  | **Day** |  |  |  |  |  |  |  | **Day** |  |  |  |  |  |  |  | **Day** |
| **Tumor** | *D1* | *D4* | *D7* | *D10* | *D13* | *D16* | *D19* | *D22* | *D1* | *D4* | *D7* | *D10* | *D13* | *D16* | *D19* | *D22* | *D22* |
| *1* | 0.3 | 0.4 | 0.5 | 0.7 | 0.7 | 1.0 | 1.1 | 1.1 | 0.3 | 0.4 | 0.5 | 0.7 | 0.8 | 1.0 | 1.2 | 1.3 | 0.629 |
| *2* | 0.3 | 0.5 | 0.7 | 0.8 | 0.9 | 1.0 | 1.0 | 1.1 | 0.3 | 0.5 | 0.7 | 0.8 | 0.9 | 1.0 | 1.2 | 1.4 | 1.215 |
| *3* | 0.3 | 0.5 | 0.7 | 1.0 | 1.1 | 1.2 | 1.3 | 1.4 | 0.4 | 0.6 | 0.8 | 1.1 | 1.2 | 1.2 | 1.3 | 1.4 | 1.252 |
| *4* | 0.3 | 0.4 | 0.5 | 0.6 | 0.7 | 0.9 | 1.0 | 1.1 | 0.3 | 0.4 | 0.6 | 0.7 | 0.8 | 0.9 | 1.1 | 1.1 | 0.522 |
| *5* | 0.2 | 0.2 | 0.3 | 0.4 | 0.4 | 0.5 | 0.5 | 0.6 | 0.3 | 0.4 | 0.5 | 0.5 | 0.6 | 0.7 | 0.7 | 0.8 | 0.412 |
| *6* | 0.2 | 0.3 | 0.5 | 0.7 | 0.9 | 1.0 | 1.1 | 1.2 | 0.3 | 0.5 | 0.7 | 0.8 | 1.0 | 1.1 | 1.2 | 1.4 | 1.141 |
| **Mean** | 0.267 | 0.383 | 0.533 | 0.700 | 0.783 | 0.933 | 1.000 | 1.083 | 0.317 | 0.467 | 0.633 | 0.767 | 0.883 | 0.983 | 1.117 | 1.233 | 0.862 |
| **SD** | 0.052 | 0.117 | 0.151 | 0.200 | 0.240 | 0.234 | 0.268 | 0.264 | 0.041 | 0.082 | 0.121 | 0.197 | 0.204 | 0.172 | 0.214 | 0.242 | 0.381 |
| **SE** | 0.021 | 0.048 | 0.061 | 0.082 | 0.098 | 0.095 | 0.110 | 0.108 | 0.017 | 0.033 | 0.049 | 0.080 | 0.083 | 0.070 | 0.087 | 0.099 | 0.156 |

| **T24 Scr** | | | | | | | | | | | | | | | | | |
| --- | --- | --- | --- | --- | --- | --- | --- | --- | --- | --- | --- | --- | --- | --- | --- | --- | --- |
| **Olaparib** | | | | | | | | | | | | | | | | | |
|  | *Width* | | | | | | | | *Length* | | | | | | | | *Net Weight* |
|  | **Day** |  |  |  |  |  |  |  | **Day** |  |  |  |  |  |  |  | **Day** |
| **Tumor** | *D1* | *D4* | *D7* | *D10* | *D13* | *D16* | *D19* | *D22* | *D1* | *D4* | *D7* | *D10* | *D13* | *D16* | *D19* | *D22* | *D22* |
| *1* | 0.3 | 0.5 | 0.6 | 0.6 | 0.8 | 0.9 | 0.9 | 0.9 | 0.3 | 0.6 | 0.8 | 0.8 | 1.1 | 1.1 | 1.3 | 1.4 | 0.287 |
| *2* | 0.2 | 0.4 | 0.6 | 0.4 | 0.5 | 0.5 | 0.5 | 0.5 | 0.3 | 0.6 | 0.7 | 0.4 | 0.5 | 0.5 | 0.7 | 0.8 | 0.177 |
| *3* | 0.1 | 0.2 | 0.5 | 0.4 | 0.7 | 0.9 | 0.9 | 1.1 | 0.2 | 0.2 | 0.5 | 0.5 | 0.8 | 0.9 | 1.0 | 1.3 | 0.192 |
| *4* | 0.2 | 0.5 | 0.8 | 0.8 | 0.8 | 0.9 | 1.2 | 1.2 | 0.3 | 0.6 | 0.9 | 1.0 | 1.6 | 1.6 | 1.8 | 1.8 | 0.785 |
| *5* | 0.3 | 0.6 | 0.6 | 0.7 | 0.9 | 0.9 | 0.9 | 0.9 | 0.3 | 0.6 | 0.8 | 0.8 | 1.2 | 1.3 | 1.4 | 1.6 | 0.475 |
| *6* | 0.2 | 0.4 | 0.4 | 0.4 | 0.4 | 0.4 | 0.4 | 0.4 | 0.2 | 0.4 | 0.4 | 0.4 | 0.4 | 0.6 | 0.6 | 0.6 | 0.564 |
| **Mean** | 0.217 | 0.433 | 0.583 | 0.550 | 0.683 | 0.750 | 0.800 | 0.833 | 0.267 | 0.500 | 0.683 | 0.650 | 0.933 | 1.000 | 1.133 | 1.250 | 0.413 |
| **SD** | 0.075 | 0.137 | 0.133 | 0.176 | 0.194 | 0.235 | 0.297 | 0.320 | 0.052 | 0.167 | 0.194 | 0.251 | 0.455 | 0.420 | 0.455 | 0.464 | 0.239 |
| **SE** | 0.031 | 0.056 | 0.054 | 0.072 | 0.079 | 0.096 | 0.121 | 0.131 | 0.021 | 0.068 | 0.079 | 0.102 | 0.186 | 0.171 | 0.186 | 0.189 | 0.097 |
| **Vehicle** | | | | | | | | | | | | | | | | | |
|  | *Width* | | | | | | | | *Length* | | | | | | | | *Net Weight* |
|  | **Day** |  |  |  |  |  |  |  | **Day** |  |  |  |  |  |  |  | **Day** |
| **Tumor** | *D1* | *D4* | *D7* | *D10* | *D13* | *D16* | *D19* | *D22* | *D1* | *D4* | *D7* | *D10* | *D13* | *D16* | *D19* | *D22* | *D22* |
| *1* | 0.1 | 0.3 | 0.3 | 0.4 | 0.5 | 0.8 | 1.0 | 1.2 | 0.2 | 0.3 | 0.4 | 0.4 | 0.6 | 0.8 | 1.1 | 1.2 | 0.852 |
| *2* | 0.3 | 0.6 | 0.8 | 0.9 | 0.9 | 0.9 | 1.0 | 1.0 | 0.3 | 0.6 | 1.0 | 1.0 | 1.0 | 1.1 | 1.2 | 1.3 | 0.517 |
| *3* | 0.3 | 0.6 | 0.8 | 1.0 | 1.0 | 1.1 | 1.2 | 1.2 | 0.4 | 0.6 | 0.8 | 1.0 | 1.1 | 1.2 | 1.3 | 1.4 | 1.348 |
| *4* | 0.3 | 0.6 | 0.7 | 0.8 | 1.4 | 1.4 | 1.3 | 1.6 | 0.5 | 1.0 | 1.2 | 1.4 | 1.6 | 1.8 | 1.8 | 1.8 | 1.945 |
| *5* | 0.2 | 0.4 | 0.5 | 0.6 | 0.9 | 0.9 | 0.9 | 0.9 | 0.3 | 0.5 | 0.6 | 0.8 | 0.9 | 0.9 | 1.0 | 1.2 | 0.905 |
| *6* | 0.2 | 0.3 | 0.5 | 0.7 | 0.9 | 1.1 | 1.1 | 1.1 | 0.2 | 0.3 | 0.5 | 0.9 | 1.5 | 1.5 | 1.5 | 1.5 | 0.627 |
| **Mean** | 0.233 | 0.467 | 0.600 | 0.733 | 0.933 | 1.033 | 1.083 | 1.167 | 0.317 | 0.550 | 0.750 | 0.917 | 1.117 | 1.217 | 1.317 | 1.400 | 1.032 |
| **SD** | 0.082 | 0.151 | 0.200 | 0.216 | 0.288 | 0.216 | 0.147 | 0.242 | 0.117 | 0.259 | 0.308 | 0.325 | 0.376 | 0.376 | 0.293 | 0.228 | 0.531 |
| **SE** | 0.033 | 0.061 | 0.082 | 0.088 | 0.117 | 0.088 | 0.060 | 0.099 | 0.048 | 0.106 | 0.126 | 0.133 | 0.154 | 0.154 | 0.119 | 0.093 | 0.217 |

| **T24 KD1** | | | | | | | | | | | | | | | | | |
| --- | --- | --- | --- | --- | --- | --- | --- | --- | --- | --- | --- | --- | --- | --- | --- | --- | --- |
| **Olaparib** | | | | | | | | | | | | | | | | | |
|  | *Width* | | | | | | | | *Length* | | | | | | | | *Net Weight* |
|  | **Day** |  |  |  |  |  |  |  | **Day** |  |  |  |  |  |  |  | **Day** |
| **Tumor** | *D1* | *D4* | *D7* | *D10* | *D13* | *D16* | *D19* | *D22* | *D1* | *D4* | *D7* | *D10* | *D13* | *D16* | *D19* | *D22* | *D22* |
| *1* | 0.3 | 0.4 | 0.4 | 0.4 | 0.3 | 0.3 | 0.3 | 0.3 | 0.4 | 0.5 | 0.5 | 0.6 | 0.6 | 0.7 | 0.8 | 0.7 | 0.080 |
| *2* | 0.3 | 0.3 | 0.3 | 0.4 | 0.5 | 0.5 | 0.5 | 0.4 | 0.3 | 0.5 | 0.5 | 0.8 | 1.0 | 1.2 | 1.3 | 1.3 | 0.320 |
| *3* | 0.2 | 0.2 | 0.2 | 0.2 | 0.2 | 0.2 | 0.2 | 0.2 | 0.3 | 0.3 | 0.2 | 0.2 | 0.2 | 0.2 | 0.2 | 0.2 | 0.065 |
| *4* | 0.1 | 0.2 | 0.2 | 0.3 | 0.3 | 0.3 | 0.3 | 0.7 | 0.1 | 0.2 | 0.2 | 0.3 | 0.3 | 0.3 | 0.3 | 0.7 | 0.075 |
| *5* | 0.2 | 0.4 | 0.4 | 0.4 | 0.6 | 0.5 | 0.8 | 0.5 | 0.2 | 0.5 | 0.5 | 0.6 | 0.8 | 0.5 | 0.8 | 0.7 | 0.074 |
| *6* | 0.2 | 0.5 | 0.5 | 0.5 | 0.6 | 0.6 | 0.7 | 0.6 | 0.3 | 0.6 | 0.6 | 0.7 | 0.7 | 0.6 | 0.9 | 0.8 | 0.122 |
| *7* | 0.2 | 0.2 | 0.3 | 0.3 | 0.5 | 0.4 | 0.6 | 0.6 | 0.3 | 0.4 | 0.5 | 0.6 | 0.8 | 0.8 | 0.9 | 0.9 | 0.054 |
| *8* | 0.2 | 0.3 | 0.3 | 0.3 | 0.4 | 0.4 | 0.4 | 0.4 | 0.2 | 0.3 | 0.3 | 0.4 | 0.4 | 0.4 | 0.4 | 0.4 | 0.039 |
| **Mean** | 0.213 | 0.313 | 0.325 | 0.350 | 0.425 | 0.400 | 0.475 | 0.463 | 0.263 | 0.413 | 0.413 | 0.525 | 0.600 | 0.588 | 0.700 | 0.713 | 0.104 |
| **SD** | 0.064 | 0.113 | 0.104 | 0.093 | 0.149 | 0.131 | 0.212 | 0.169 | 0.092 | 0.136 | 0.155 | 0.205 | 0.278 | 0.318 | 0.370 | 0.327 | 0.091 |
| **SE** | 0.023 | 0.040 | 0.037 | 0.033 | 0.053 | 0.046 | 0.075 | 0.060 | 0.032 | 0.048 | 0.055 | 0.073 | 0.098 | 0.113 | 0.131 | 0.116 | 0.032 |
| **Vehicle** | | | | | | | | | | | | | | | | | |
|  | *Width* | | | | | | | | *Length* | | | | | | | | *Net Weight* |
|  | **Day** |  |  |  |  |  |  |  | **Day** |  |  |  |  |  |  |  | **Day** |
| **Tumor** | *D1* | *D4* | *D7* | *D10* | *D13* | *D16* | *D19* | *D22* | *D1* | *D4* | *D7* | *D10* | *D13* | *D16* | *D19* | *D22* | *D22* |
| *1* | 0.3 | 0.5 | 0.6 | 0.6 | 0.6 | 0.6 | 0.6 | 0.6 | 0.5 | 0.7 | 0.7 | 0.7 | 0.8 | 0.8 | 0.8 | 0.8 | 0.112 |
| *2* | 0.2 | 0.4 | 0.6 | 0.7 | 0.8 | 0.8 | 0.9 | 1.0 | 0.4 | 0.7 | 0.9 | 1.0 | 1.1 | 1.2 | 1.2 | 1.4 | 1.051 |
| *3* | 0.2 | 0.4 | 0.5 | 0.5 | 0.5 | 0.5 | 0.7 | 0.7 | 0.2 | 0.4 | 0.5 | 0.5 | 0.5 | 0.6 | 0.7 | 0.8 | 0.283 |
| *4* | 0.2 | 0.3 | 0.4 | 0.4 | 0.6 | 0.8 | 0.9 | 0.9 | 0.3 | 0.5 | 0.7 | 0.8 | 1.0 | 1.2 | 1.3 | 1.4 | 0.842 |
| *5* | 0.3 | 0.4 | 0.4 | 0.5 | 0.7 | 1.0 | 1.1 | 1.1 | 0.3 | 0.5 | 0.6 | 0.8 | 0.9 | 1.1 | 1.2 | 1.3 | 1.093 |
| *6* | 0.2 | 0.2 | 0.3 | 0.4 | 0.6 | 0.7 | 0.8 | 0.8 | 0.2 | 0.3 | 0.3 | 0.5 | 0.6 | 0.8 | 0.8 | 1.0 | 0.371 |
| *7* | 0.1 | 0.2 | 0.4 | 0.5 | 0.5 | 0.6 | 0.7 | 0.7 | 0.3 | 0.4 | 0.5 | 0.5 | 0.6 | 0.6 | 0.7 | 0.8 | 0.178 |
| **Mean** | 0.214 | 0.343 | 0.457 | 0.514 | 0.614 | 0.714 | 0.814 | 0.829 | 0.314 | 0.500 | 0.600 | 0.686 | 0.786 | 0.900 | 0.957 | 1.071 | 0.562 |
| **SD** | 0.069 | 0.113 | 0.113 | 0.107 | 0.107 | 0.168 | 0.168 | 0.180 | 0.107 | 0.153 | 0.191 | 0.195 | 0.227 | 0.265 | 0.264 | 0.287 | 0.421 |
| **SE** | 0.026 | 0.043 | 0.043 | 0.040 | 0.040 | 0.063 | 0.063 | 0.068 | 0.040 | 0.058 | 0.072 | 0.074 | 0.086 | 0.100 | 0.100 | 0.108 | 0.159 |

| **H1437 Scr** | | | | | | | | | | | | | | | | | |
| --- | --- | --- | --- | --- | --- | --- | --- | --- | --- | --- | --- | --- | --- | --- | --- | --- | --- |
| **Olaparib** | | | | | | | | | | | | | | | | | |
|  | *Width* | | | | | | | | *Length* | | | | | | | | *Net Weight* |
|  | **Day** |  |  |  |  |  |  |  | **Day** |  |  |  |  |  |  |  | **Day** |
| **Tumor** | *D1* | *D4* | *D7* | *D10* | *D13* | *D16* | *D19* | *D22* | *D1* | *D4* | *D7* | *D10* | *D13* | *D16* | *D19* | *D22* | *D22* |
| *1* | 0.3 | 0.6 | 0.7 | 0.8 | 0.8 | 0.8 | 0.8 | 0.8 | 0.4 | 0.8 | 1.1 | 1.3 | 1.3 | 1.5 | 1.6 | 1.7 | 0.554 |
| *2* | 0.2 | 0.4 | 0.6 | 0.6 | 0.6 | 0.6 | 0.8 | 0.9 | 0.3 | 0.6 | 0.8 | 0.9 | 0.9 | 1.0 | 1.0 | 1.0 | 0.362 |
| *3* | 0.1 | 0.2 | 0.4 | 0.5 | 0.7 | 0.8 | 0.9 | 1.0 | 0.3 | 0.4 | 0.6 | 0.7 | 0.7 | 0.8 | 0.9 | 1.1 | 0.638 |
| *4* | 0.1 | 0.3 | 0.5 | 0.7 | 0.7 | 0.8 | 0.8 | 0.9 | 0.3 | 0.6 | 0.8 | 1.0 | 1.1 | 1.1 | 1.1 | 1.2 | 0.646 |
| *5* | 0.2 | 0.4 | 0.6 | 0.6 | 0.6 | 0.8 | 0.8 | 0.8 | 0.3 | 0.5 | 0.6 | 0.8 | 0.8 | 1.2 | 1.1 | 1.1 | 0.389 |
| *6* | 0.3 | 0.4 | 0.5 | 0.6 | 0.7 | 0.9 | 0.9 | 0.9 | 0.4 | 0.8 | 1.1 | 1.3 | 1.3 | 1.6 | 1.7 | 1.8 | 1.207 |
| *7* | 0.2 | 0.4 | 0.5 | 0.6 | 0.6 | 0.9 | 0.9 | 0.9 | 0.3 | 0.7 | 1.0 | 1.2 | 1.3 | 1.5 | 1.5 | 1.7 | 1.233 |
| *8* | 0.3 | 0.4 | 0.5 | 0.6 | 0.8 | 1.3 | 1.3 | 1.3 | 0.4 | 0.8 | 1.0 | 1.2 | 1.3 | 1.7 | 1.5 | 1.6 | 1.210 |
| **Mean** | 0.213 | 0.388 | 0.538 | 0.625 | 0.688 | 0.863 | 0.900 | 0.938 | 0.338 | 0.650 | 0.875 | 1.050 | 1.088 | 1.300 | 1.300 | 1.400 | 0.780 |
| **SD** | 0.083 | 0.113 | 0.092 | 0.089 | 0.083 | 0.200 | 0.169 | 0.160 | 0.052 | 0.151 | 0.205 | 0.233 | 0.253 | 0.321 | 0.307 | 0.330 | 0.376 |
| **SE** | 0.030 | 0.040 | 0.032 | 0.031 | 0.030 | 0.071 | 0.060 | 0.056 | 0.018 | 0.053 | 0.073 | 0.082 | 0.090 | 0.113 | 0.109 | 0.116 | 0.133 |
| **Vehicle** | | | | | | | | | | | | | | | | | |
|  | *Width* | | | | | | | | *Length* | | | | | | | | *Net Weight* |
|  | **Day** |  |  |  |  |  |  |  | **Day** |  |  |  |  |  |  |  | **Day** |
| **Tumor** | *D1* | *D4* | *D7* | *D10* | *D13* | *D16* | *D19* | *D22* | *D1* | *D4* | *D7* | *D10* | *D13* | *D16* | *D19* | *D22* | *D22* |
| *1* | 0.3 | 0.6 | 0.8 | 0.9 | 0.9 | 0.9 | 1.0 | 1.0 | 0.3 | 0.6 | 1.0 | 1.0 | 1.0 | 1.1 | 1.2 | 1.3 | 0.517 |
| *2* | 0.3 | 0.6 | 0.8 | 1.0 | 1.0 | 1.1 | 1.2 | 1.2 | 0.4 | 0.6 | 0.8 | 1.0 | 1.1 | 1.2 | 1.3 | 1.4 | 1.348 |
| *3* | 0.2 | 0.3 | 0.6 | 0.7 | 0.8 | 0.8 | 0.9 | 0.9 | 0.3 | 0.5 | 0.7 | 0.8 | 0.8 | 0.9 | 1.0 | 1.1 | 0.417 |
| *4* | 0.1 | 0.2 | 0.3 | 0.5 | 0.7 | 0.6 | 0.6 | 0.7 | 0.2 | 0.3 | 0.4 | 0.6 | 0.7 | 1.2 | 1.3 | 1.4 | 0.401 |
| *5* | 0.2 | 0.3 | 0.4 | 0.6 | 0.7 | 0.8 | 0.9 | 0.9 | 0.3 | 0.3 | 0.5 | 0.6 | 0.8 | 1.0 | 1.2 | 1.3 | 0.415 |
| *6* | 0.2 | 0.4 | 0.5 | 0.6 | 0.9 | 0.9 | 0.9 | 0.9 | 0.3 | 0.5 | 0.6 | 0.8 | 0.9 | 0.9 | 1.0 | 1.2 | 0.905 |
| *7* | 0.2 | 0.3 | 0.5 | 0.7 | 0.9 | 1.1 | 1.1 | 1.1 | 0.2 | 0.3 | 0.5 | 0.9 | 1.5 | 1.5 | 1.5 | 1.5 | 0.627 |
| **Mean** | 0.214 | 0.386 | 0.557 | 0.714 | 0.843 | 0.886 | 0.943 | 0.957 | 0.286 | 0.443 | 0.643 | 0.814 | 0.971 | 1.114 | 1.214 | 1.314 | 0.661 |
| **SD** | 0.069 | 0.157 | 0.190 | 0.177 | 0.113 | 0.177 | 0.190 | 0.162 | 0.069 | 0.140 | 0.207 | 0.168 | 0.269 | 0.212 | 0.177 | 0.135 | 0.351 |
| **SE** | 0.026 | 0.059 | 0.072 | 0.067 | 0.043 | 0.067 | 0.072 | 0.061 | 0.026 | 0.053 | 0.078 | 0.063 | 0.102 | 0.080 | 0.067 | 0.051 | 0.133 |

| **H1437 KD1** | | | | | | | | | | | | | | | | | |
| --- | --- | --- | --- | --- | --- | --- | --- | --- | --- | --- | --- | --- | --- | --- | --- | --- | --- |
| **Olaparib** | | | | | | | | | | | | | | | | | |
|  | *Width* | | | | | | | | *Length* | | | | | | | | *Net Weight* |
|  | **Day** |  |  |  |  |  |  |  | **Day** |  |  |  |  |  |  |  | **Day** |
| **Tumor** | *D1* | *D4* | *D7* | *D10* | *D13* | *D16* | *D19* | *D22* | *D1* | *D4* | *D7* | *D10* | *D13* | *D16* | *D19* | *D22* | *D22* |
| *1* | 0.3 | 0.5 | 0.6 | 0.7 | 0.7 | 0.6 | 0.8 | 0.8 | 0.3 | 0.5 | 0.6 | 0.7 | 0.8 | 0.7 | 0.9 | 1.1 | 0.578 |
| *2* | 0.3 | 0.5 | 0.6 | 0.7 | 0.6 | 0.6 | 0.7 | 0.8 | 0.4 | 0.7 | 0.8 | 0.9 | 0.8 | 0.8 | 1.0 | 1.2 | 0.988 |
| *3* | 0.2 | 0.3 | 0.5 | 0.6 | 0.6 | 0.5 | 0.7 | 0.7 | 0.3 | 0.4 | 0.7 | 0.8 | 0.9 | 0.7 | 0.7 | 0.8 | 0.407 |
| *4* | 0.1 | 0.3 | 0.4 | 0.4 | 0.5 | 0.4 | 0.5 | 0.5 | 0.3 | 0.4 | 0.5 | 0.6 | 0.5 | 0.5 | 0.6 | 0.7 | 0.189 |
| *5* | 0.3 | 0.3 | 0.4 | 0.4 | 0.4 | 0.4 | 0.4 | 0.6 | 0.3 | 0.3 | 0.4 | 0.5 | 0.5 | 0.5 | 0.5 | 0.6 | 0.330 |
| *6* | 0.4 | 0.4 | 0.5 | 0.6 | 0.6 | 0.6 | 0.6 | 0.6 | 0.4 | 0.5 | 0.5 | 0.6 | 0.6 | 0.7 | 0.7 | 0.7 | 0.286 |
| *7* | 0.3 | 0.3 | 0.3 | 0.4 | 0.4 | 0.4 | 0.4 | 0.4 | 0.3 | 0.3 | 0.4 | 0.4 | 0.4 | 0.4 | 0.4 | 0.4 | 0.043 |
| *8* | 0.2 | 0.2 | 0.3 | 0.3 | 0.3 | 0.3 | 0.3 | 0.4 | 0.2 | 0.3 | 0.3 | 0.4 | 0.4 | 0.4 | 0.4 | 0.4 | 0.086 |
| **Mean** | 0.263 | 0.350 | 0.450 | 0.513 | 0.513 | 0.475 | 0.550 | 0.600 | 0.313 | 0.425 | 0.525 | 0.613 | 0.613 | 0.588 | 0.650 | 0.738 | 0.363 |
| **SD** | 0.092 | 0.107 | 0.120 | 0.155 | 0.136 | 0.116 | 0.177 | 0.160 | 0.064 | 0.139 | 0.167 | 0.181 | 0.196 | 0.155 | 0.220 | 0.292 | 0.306 |
| **SE** | 0.032 | 0.038 | 0.042 | 0.055 | 0.048 | 0.041 | 0.063 | 0.057 | 0.023 | 0.049 | 0.059 | 0.064 | 0.069 | 0.055 | 0.078 | 0.103 | 0.108 |
| **Vehicle** | | | | | | | | | | | | | | | | | |
|  | *Width* | | | | | | | | *Length* | | | | | | | | *Net Weight* |
|  | **Day** |  |  |  |  |  |  |  | **Day** |  |  |  |  |  |  |  | **Day** |
| **Tumor** | *D1* | *D4* | *D7* | *D10* | *D13* | *D16* | *D19* | *D22* | *D1* | *D4* | *D7* | *D10* | *D13* | *D16* | *D19* | *D22* | *D22* |
| *1* | 0.3 | 0.5 | 0.7 | 1.0 | 1.1 | 1.2 | 1.2 | 1.2 | 0.4 | 0.7 | 0.9 | 1.1 | 1.3 | 1.3 | 1.3 | 1.4 | 0.836 |
| *2* | 0.2 | 0.4 | 0.5 | 0.6 | 0.8 | 1.0 | 1.1 | 1.2 | 0.2 | 0.4 | 0.7 | 0.9 | 1.0 | 1.1 | 1.1 | 1.3 | 0.732 |
| *3* | 0.2 | 0.2 | 0.2 | 0.5 | 0.6 | 0.6 | 0.8 | 0.9 | 0.2 | 0.2 | 0.2 | 0.5 | 0.6 | 0.7 | 0.8 | 1.0 | 0.443 |
| *4* | 0.1 | 0.2 | 0.3 | 0.5 | 0.6 | 0.7 | 0.8 | 0.9 | 0.3 | 0.5 | 0.9 | 1.2 | 1.2 | 1.2 | 1.3 | 1.4 | 0.631 |
| *5* | 0.2 | 0.4 | 0.7 | 0.9 | 0.9 | 0.9 | 0.9 | 1.0 | 0.3 | 0.6 | 0.9 | 1.2 | 1.3 | 1.3 | 1.3 | 1.3 | 1.303 |
| *6* | 0.2 | 0.3 | 0.4 | 0.6 | 0.6 | 0.6 | 0.7 | 0.7 | 0.4 | 0.7 | 1.0 | 1.3 | 1.4 | 1.4 | 1.4 | 1.5 | 0.828 |
| *7* | 0.1 | 0.2 | 0.4 | 0.5 | 0.5 | 0.5 | 0.5 | 0.7 | 0.3 | 0.5 | 0.7 | 0.9 | 0.9 | 1.0 | 1.1 | 1.2 | 0.459 |
| *8* | 0.1 | 0.3 | 0.4 | 0.6 | 0.6 | 0.6 | 0.7 | 0.7 | 0.3 | 0.6 | 0.9 | 1.2 | 1.3 | 1.3 | 1.3 | 1.3 | 0.550 |
| **Mean** | 0.175 | 0.313 | 0.450 | 0.650 | 0.713 | 0.763 | 0.838 | 0.913 | 0.300 | 0.525 | 0.775 | 1.038 | 1.125 | 1.163 | 1.200 | 1.300 | 0.723 |
| **SD** | 0.071 | 0.113 | 0.177 | 0.193 | 0.203 | 0.245 | 0.226 | 0.210 | 0.076 | 0.167 | 0.255 | 0.262 | 0.271 | 0.226 | 0.193 | 0.151 | 0.279 |
| **SE** | 0.025 | 0.040 | 0.063 | 0.068 | 0.072 | 0.086 | 0.080 | 0.074 | 0.027 | 0.059 | 0.090 | 0.092 | 0.096 | 0.080 | 0.068 | 0.053 | 0.099 |

| **Cal-33 Scr** | | | | | | | | | | | | | | | | | |
| --- | --- | --- | --- | --- | --- | --- | --- | --- | --- | --- | --- | --- | --- | --- | --- | --- | --- |
| **Olaparib** | | | | | | | | | | | | | | | | | |
|  | *Width* | | | | | | | | *Length* | | | | | | | | *Net Weight* |
|  | **Day** |  |  |  |  |  |  |  | **Day** |  |  |  |  |  |  |  | **Day** |
| **Tumor** | *D1* | *D4* | *D7* | *D10* | *D13* | *D16* | *D19* | *D22* | *D1* | *D4* | *D7* | *D10* | *D13* | *D16* | *D19* | *D22* | *D22* |
| *1* | 0.2 | 0.3 | 0.5 | 0.6 | 0.7 | 0.7 | 0.8 | 0.9 | 0.2 | 0.3 | 0.6 | 0.8 | 0.9 | 1.0 | 1.2 | 1.4 | 0.223 |
| *2* | 0.1 | 0.3 | 0.4 | 0.6 | 0.8 | 0.9 | 1.0 | 1.2 | 0.2 | 0.3 | 0.5 | 0.6 | 0.8 | 1.0 | 1.2 | 1.2 | 0.287 |
| *3* | 0.3 | 0.5 | 0.7 | 0.6 | 0.8 | 1.0 | 1.2 | 1.4 | 0.3 | 0.6 | 0.7 | 1.0 | 1.0 | 1.2 | 1.2 | 1.4 | 0.464 |
| *4* | 0.1 | 0.1 | 0.3 | 0.6 | 0.7 | 0.8 | 1.0 | 1.1 | 0.1 | 0.2 | 0.4 | 0.6 | 1.0 | 1.5 | 1.5 | 1.6 | 0.374 |
| *5* | 0.2 | 0.3 | 0.5 | 0.7 | 0.7 | 0.7 | 0.9 | 1.0 | 0.2 | 0.3 | 0.6 | 0.7 | 0.8 | 0.8 | 0.9 | 1.0 | 0.204 |
| *6* | 0.1 | 0.1 | 0.1 | 0.4 | 0.5 | 0.5 | 0.6 | 1.0 | 0.1 | 0.3 | 0.3 | 1.0 | 1.1 | 1.2 | 1.4 | 1.8 | 0.299 |
| *7* | 0.2 | 0.5 | 0.8 | 0.7 | 0.8 | 0.8 | 1.0 | 1.1 | 0.4 | 0.5 | 0.8 | 0.8 | 0.8 | 0.9 | 1.0 | 1.2 | 0.277 |
| *8* | 0.2 | 0.3 | 0.6 | 0.7 | 0.7 | 0.7 | 0.9 | 1.1 | 0.2 | 0.4 | 0.7 | 0.7 | 0.8 | 0.8 | 1.0 | 1.2 | 0.285 |
| **Mean** | 0.175 | 0.300 | 0.488 | 0.613 | 0.713 | 0.763 | 0.925 | 1.100 | 0.213 | 0.363 | 0.575 | 0.775 | 0.900 | 1.050 | 1.175 | 1.350 | 0.301 |
| **SD** | 0.071 | 0.151 | 0.223 | 0.099 | 0.099 | 0.151 | 0.175 | 0.151 | 0.099 | 0.130 | 0.167 | 0.158 | 0.120 | 0.239 | 0.205 | 0.256 | 0.083 |
| **SE** | 0.025 | 0.053 | 0.079 | 0.035 | 0.035 | 0.053 | 0.062 | 0.053 | 0.035 | 0.046 | 0.059 | 0.056 | 0.042 | 0.085 | 0.073 | 0.091 | 0.029 |
| **Vehicle** | | | | | | | | | | | | | | | | | |
|  | *Width* | | | | | | | | *Length* | | | | | | | | *Net Weight* |
|  | **Day** |  |  |  |  |  |  |  | **Day** |  |  |  |  |  |  |  | **Day** |
| **Tumor** | *D1* | *D4* | *D7* | *D10* | *D13* | *D16* | *D19* | *D22* | *D1* | *D4* | *D7* | *D10* | *D13* | *D16* | *D19* | *D22* | *D22* |
| *1* | 0.2 | 0.4 | 0.5 | 0.8 | 0.9 | 1.0 | 1.2 | 1.4 | 0.2 | 0.5 | 0.6 | 0.8 | 0.9 | 1.0 | 1.2 | 1.4 | 0.396 |
| *2* | 0.2 | 0.4 | 0.6 | 1.0 | 1.1 | 1.0 | 1.2 | 1.4 | 0.2 | 0.4 | 0.7 | 1.1 | 1.1 | 1.1 | 1.3 | 1.5 | 0.434 |
| *3* | 0.2 | 0.3 | 0.6 | 0.8 | 1.0 | 1.1 | 1.2 | 1.3 | 0.2 | 0.4 | 0.6 | 0.9 | 1.0 | 1.1 | 1.3 | 1.4 | 0.356 |
| *4* | 0.4 | 0.6 | 0.7 | 0.9 | 0.9 | 0.9 | 1.1 | 1.3 | 0.4 | 0.6 | 0.9 | 1.1 | 1.1 | 1.2 | 1.4 | 1.6 | 0.611 |
| *5* | 0.3 | 0.5 | 0.6 | 0.6 | 0.7 | 0.8 | 1.0 | 1.2 | 0.3 | 0.6 | 0.8 | 0.8 | 0.9 | 0.9 | 1.3 | 1.6 | 0.390 |
| *6* | 0.2 | 0.4 | 0.5 | 0.6 | 0.7 | 0.9 | 1.0 | 1.2 | 0.3 | 0.8 | 0.8 | 0.7 | 1.1 | 1.1 | 1.2 | 1.3 | 0.256 |
| *7* | 0.2 | 0.4 | 0.7 | 0.9 | 0.9 | 0.9 | 1.1 | 1.4 | 0.3 | 0.4 | 0.9 | 1.0 | 1.0 | 1.1 | 1.3 | 1.6 | 0.629 |
| **Mean** | 0.243 | 0.429 | 0.600 | 0.800 | 0.886 | 0.943 | 1.114 | 1.314 | 0.271 | 0.529 | 0.757 | 0.914 | 1.014 | 1.071 | 1.286 | 1.486 | 0.439 |
| **SD** | 0.079 | 0.095 | 0.082 | 0.153 | 0.146 | 0.098 | 0.090 | 0.090 | 0.076 | 0.150 | 0.127 | 0.157 | 0.090 | 0.095 | 0.069 | 0.121 | 0.135 |
| **SE** | 0.030 | 0.036 | 0.031 | 0.058 | 0.055 | 0.037 | 0.034 | 0.034 | 0.029 | 0.057 | 0.048 | 0.059 | 0.034 | 0.036 | 0.026 | 0.046 | 0.051 |

| **Cal-33 KD1** | | | | | | | | | | | | | | | | | |
| --- | --- | --- | --- | --- | --- | --- | --- | --- | --- | --- | --- | --- | --- | --- | --- | --- | --- |
| **Olaparib** | | | | | | | | | | | | | | | | | |
|  | *Width* | | | | | | | | *Length* | | | | | | | | *Net Weight* |
|  | **Day** |  |  |  |  |  |  |  | **Day** |  |  |  |  |  |  |  | **Day** |
| **Tumor** | *D1* | *D4* | *D7* | *D10* | *D13* | *D16* | *D19* | *D22* | *D1* | *D4* | *D7* | *D10* | *D13* | *D16* | *D19* | *D22* | *D22* |
| *1* | 0.2 | 0.2 | 0.2 | 0.2 | 0.2 | 0.3 | 0.2 | 0.2 | 0.2 | 0.2 | 0.2 | 0.2 | 0.2 | 0.3 | 0.3 | 0.2 | 0.020 |
| *2* | 0.2 | 0.3 | 0.3 | 0.3 | 0.3 | 0.3 | 0.3 | 0.3 | 0.3 | 0.3 | 0.3 | 0.3 | 0.3 | 0.3 | 0.4 | 0.3 | 0.022 |
| *3* | 0.2 | 0.3 | 0.4 | 0.4 | 0.6 | 0.6 | 0.7 | 0.7 | 0.2 | 0.3 | 0.4 | 0.5 | 0.7 | 0.8 | 0.9 | 0.9 | 0.108 |
| *4* | 0.3 | 0.3 | 0.3 | 0.3 | 0.2 | 0.2 | 0.2 | 0.2 | 0.3 | 0.3 | 0.4 | 0.4 | 0.2 | 0.2 | 0.2 | 0.2 | 0.007 |
| *5* | 0.2 | 0.2 | 0.3 | 0.3 | 0.3 | 0.3 | 0.3 | 0.3 | 0.2 | 0.3 | 0.3 | 0.3 | 0.3 | 0.4 | 0.4 | 0.4 | 0.067 |
| *6* | 0.3 | 0.3 | 0.3 | 0.3 | 0.4 | 0.4 | 0.4 | 0.4 | 0.4 | 0.4 | 0.4 | 0.4 | 0.4 | 0.4 | 0.4 | 0.4 | 0.076 |
| **Mean** | 0.233 | 0.267 | 0.300 | 0.300 | 0.333 | 0.350 | 0.350 | 0.350 | 0.267 | 0.300 | 0.333 | 0.350 | 0.350 | 0.400 | 0.433 | 0.400 | 0.050 |
| **SD** | 0.052 | 0.052 | 0.063 | 0.063 | 0.151 | 0.138 | 0.187 | 0.187 | 0.082 | 0.063 | 0.082 | 0.105 | 0.187 | 0.210 | 0.242 | 0.261 | 0.040 |
| **SE** | 0.021 | 0.021 | 0.026 | 0.026 | 0.061 | 0.056 | 0.076 | 0.076 | 0.033 | 0.026 | 0.033 | 0.043 | 0.076 | 0.086 | 0.099 | 0.106 | 0.016 |
| **Vehicle** | | | | | | | | | | | | | | | | | |
|  | *Width* | | | | | | | | *Length* | | | | | | | | *Net Weight* |
|  | **Day** |  |  |  |  |  |  |  | **Day** |  |  |  |  |  |  |  | **Day** |
| **Tumor** | *D1* | *D4* | *D7* | *D10* | *D13* | *D16* | *D19* | *D22* | *D1* | *D4* | *D7* | *D10* | *D13* | *D16* | *D19* | *D22* | *D22* |
| *1* | 0.3 | 0.4 | 0.5 | 0.6 | 0.6 | 0.6 | 0.7 | 0.9 | 0.4 | 0.5 | 0.6 | 0.8 | 0.8 | 0.9 | 1.1 | 1.3 | 0.693 |
| *2* | 0.2 | 0.3 | 0.4 | 0.6 | 0.6 | 0.6 | 0.6 | 0.8 | 0.3 | 0.5 | 0.6 | 0.8 | 0.9 | 1.0 | 1.0 | 1.2 | 0.302 |
| *3* | 0.6 | 0.7 | 0.8 | 0.8 | 0.9 | 0.9 | 0.9 | 1.1 | 0.6 | 0.7 | 0.8 | 0.9 | 1.0 | 1.1 | 1.1 | 1.4 | 1.655 |
| *4* | 0.2 | 0.4 | 0.5 | 0.5 | 0.5 | 0.5 | 0.6 | 0.7 | 0.2 | 0.4 | 0.6 | 0.7 | 0.8 | 0.8 | 0.9 | 1.3 | 0.332 |
| *5* | 0.4 | 0.4 | 0.5 | 0.5 | 0.6 | 0.6 | 0.7 | 0.8 | 0.4 | 0.5 | 0.8 | 0.9 | 1.0 | 1.1 | 1.2 | 1.2 | 0.235 |
| *6* | 0.3 | 0.3 | 0.4 | 0.4 | 0.5 | 0.5 | 0.7 | 0.7 | 0.3 | 0.3 | 0.4 | 0.5 | 0.7 | 0.9 | 1.1 | 1.2 | 0.290 |
| **Mean** | 0.333 | 0.417 | 0.517 | 0.567 | 0.617 | 0.617 | 0.700 | 0.833 | 0.367 | 0.483 | 0.633 | 0.767 | 0.867 | 0.967 | 1.067 | 1.267 | 0.584 |
| **SD** | 0.151 | 0.147 | 0.147 | 0.137 | 0.147 | 0.147 | 0.110 | 0.151 | 0.137 | 0.133 | 0.151 | 0.151 | 0.121 | 0.121 | 0.103 | 0.082 | 0.550 |
| **SE** | 0.061 | 0.060 | 0.060 | 0.056 | 0.060 | 0.060 | 0.045 | 0.061 | 0.056 | 0.054 | 0.061 | 0.061 | 0.049 | 0.049 | 0.042 | 0.033 | 0.224 |

**Appendix Table S4.** P values associated with Figure 8D.

| **HTB9** | | | | | |
| --- | --- | --- | --- | --- | --- |
| **HTB9_Scr** | | **HTB9_X44** | | **HTB9_Scr *vs* HTB9_X44** | |
| **Olaparib vs Vehicle** | | **Olaparib vs Vehicle** | | **Olaparib vs Olaparib** | |
| **Day1** | 0.424 | **Day1** | 0.037 | **Day1** | 0.390 |
| **Day4** | 0.052 | **Day4** | 0.002 | **Day4** | 0.002 |
| **Day7** | 0.010 | **Day7** | 0.002 | **Day7** | 0.002 |
| **Day10** | 0.007 | **Day10** | 0.002 | **Day10** | 0.002 |
| **Day13** | 0.007 | **Day13** | 0.006 | **Day13** | 0.005 |
| **Day16** | 0.008 | **Day16** | 0.003 | **Day16** | 0.009 |
| **Day19** | 0.008 | **Day19** | 0.003 | **Day19** | 0.003 |
| **Day22** | 0.012 | **Day22** | 0.003 | **Day22** | 0.004 |
| **Net Weight** | 0.153 | **Net Weight** | 0.003 | **Net Weight** | 0.002 |
| **T84** | | | | | |
| **T84 Scr** | | **T84 X44** | | **T84 Scr *vs* T84 X44** | |
| **Olaparib vs Vehicle** | | **Olaparib vs Vehicle** | | **Olaparib vs Olaparib** | |
| **Day1** | 0.514 | **Day1** | 0.813 | **Day1** | 0.894 |
| **Day4** | 0.627 | **Day4** | 0.449 | **Day4** | 0.135 |
| **Day7** | 0.688 | **Day7** | 0.042 | **Day7** | 0.010 |
| **Day10** | 0.125 | **Day10** | 0.032 | **Day10** | 0.121 |
| **Day13** | 0.109 | **Day13** | 0.056 | **Day13** | 0.045 |
| **Day16** | 0.199 | **Day16** | 0.006 | **Day16** | 0.033 |
| **Day19** | 0.128 | **Day19** | 0.024 | **Day19** | 0.039 |
| **Day22** | 0.150 | **Day22** | 0.003 | **Day22** | 0.053 |
| **Net Weight** | 0.016 | **Net Weight** | 0.005 | **Net Weight** | 0.007 |
| **H1437** | | | | | |
| **H1437 Scr** | | **H1437 X44** | | **H1437 Scr *vs* H1437 X44** | |
| **Olaparib vs Vehicle** | | **Olaparib vs Vehicle** | | **Olaparib vs Olaparib** | |
| **Day1** | 0.629 | **Day1** | 0.098 | **Day1** | 0.668 |
| **Day4** | 0.324 | **Day4** | 0.874 | **Day4** | 0.155 |
| **Day7** | 0.487 | **Day7** | 0.430 | **Day7** | 0.027 |
| **Day10** | 0.772 | **Day10** | 0.036 | **Day10** | 0.024 |
| **Day13** | 0.271 | **Day13** | 0.007 | **Day13** | 0.004 |
| **Day16** | 0.817 | **Day16** | 0.004 | **Day16** | 0.001 |
| **Day19** | 0.728 | **Day19** | 0.003 | **Day19** | 0.001 |
| **Day22** | 0.862 | **Day22** | 0.005 | **Day22** | 0.001 |
| **Net Weight** | 0.643 | **Net Weight** | 0.016 | **Net Weight** | 0.021 |
| **Cal33** | | | | | |
| **Cal33 Scr** | | **Cal33 X44** | | **Cal33 Scr *vs* Cal33 X44** | |
| **Olaparib vs Vehicle** | | **Olaparib vs Vehicle** | | **Olaparib vs Olaparib** | |
| **Day1** | 0.119 | **Day1** | 0.140 | **Day1** | 0.138 |
| **Day4** | 0.036 | **Day4** | 0.011 | **Day4** | 0.590 |
| **Day7** | 0.181 | **Day7** | 0.005 | **Day7** | 0.038 |
| **Day10** | 0.032 | **Day10** | 0.005 | **Day10** | 0.002 |
| **Day13** | 0.009 | **Day13** | 0.010 | **Day13** | 0.002 |
| **Day16** | 0.042 | **Day16** | 0.010 | **Day16** | 0.002 |
| **Day19** | 0.015 | **Day19** | 0.010 | **Day19** | 0.002 |
| **Day22** | 0.009 | **Day22** | 0.004 | **Day22** | 0.002 |
| **Net Weight** | 0.049 | **Net Weight** | 0.004 | **Net Weight** | 0.002 |

**Appendix Table S5.** Mutation detection primers

| 1^st^ PCR | Sequence |  | Nested | Sequence |
| --- | --- | --- | --- | --- |
| NTF | ATGTCGTCGGAGGAGGACAAG |  | NTFN | AGCAGCCGCAGCCGCCGCCAC |
| NTR | ATCTGGAAGCCCAACCAGACT |  | NTRN | AGCTTGATCATCTGAAGCTGT |
|  |  |  |  |  |
| PHD1F | ATGATCAAGCTGGTAAACTGT |  | PHD1FN | ATGAACTCAGTCTGGTTGGGG |
| PHD1R | TAGCATCTTGCTATCTTCTCC |  | PHD1RN | TCAGGACATTGCCAACCTGCA |
|  |  |  |  |  |
| PHD2FB | AGGTTGGCAATGTCCTGAGTG |  | PHD2FN | ATATAGCGGTTACTCCATTAA |
| PHD2RA | TTCTTTGAGCTGAGTATCCAG |  | PHD2RC | ACAATACATGCAGATATACTC |
|  |  |  |  |  |
| SET1F | ATCCTGTGCCCAAAGACTATC |  | SET1FN | ATGAAGAAGGTGATGGATGA |
| SET01R | TCCTGATGACAAACACTGGGC |  | SET01RN | TCAATGGAGCACAGGTAGCGG |
|  |  |  |  |  |
| SET02CF | ATCTTCCACACAATTGGTCAG |  | SET02RN | ACATTGGATTTCCATTCAGTT |
| SET02CR | TACTCAATGACCATGGTGTGT |  | SET02FN | ATGAAGCCAGCCGCCTGTACT |
|  |  |  |  |  |
| SET03F | AGTCACTGGAGAACTGAACGC |  | SET03RN | TCACAGCTCCACAGTGACACG |
| SET03R | TCAGTTCATCCACTTCCGGCA |  | SET03FN | AGTCATCGCAGTACCGGAAGA |

**Appendix Table S6.** Quantitative RT-PCR oligonucleotides used

| Gene | Primer | Sequence | |
| --- | --- | --- | --- |
| MLL3/KMT2C | Forward Primer | CCATGCTGTCCTCATCTTCC | |
|  | Reverse Primer | AGCCGCAGACAAAAGACCT | |
|  |  |  | |
| HPRT1 | Forward Primer | TGACACTGGCAAAACAATGCA | |
|  | Reverse Primer | GGTCCTTTTCACCAGCAAGCT | |
|  |  |  | |
| β-actin | Forward Primer | TTCCTGGGCATGGAGTC | |
|  | Reverse Primer | CAGGTCTTTGCGGATGTC | |
|  |  |  | |
| [ATM](http://www.ensembl.org/Homo_sapiens/Transcript/Sequence_cDNA?db=core;g=ENSG00000149311;r=11:108222484-108369102;t=ENST00000527805) | Forward Primer | ATCTGCTGCCGTCAACTAGAA | |
|  | Reverse Primer | GATCTCGAATCAGGCGCTTAAA | |
|  |  |  | |
| [ATR](http://www.ensembl.org/Homo_sapiens/Transcript/Sequence_cDNA?db=core;g=ENSG00000175054;r=3:142449235-142578826;t=ENST00000350721) | Forward Primer | ACCTCAGCAGTAATAGTGATGGA | |
|  | Reverse Primer | GGCCACTGTATTCAAGGGAAAT | |
|  |  |  | |
| CHEK2 | Forward Primer | TCTGGCTTTAAGTCACGGTGTA | |
|  | Reverse Primer | AGTGGTGGGGAATAAACGCC | |
|  |  |  | |
| BRCA1 | Forward Primer | GGCTATCCTCTCAGAGTGACATTT | |
|  | Reverse Primer | GCTTTAGGTTATGTTGCATGGT | |
|  |  |  | |
| CHEK1 | Forward Primer | CCTCATCCCTCACCACAGAT | |
|  | Reverse Primer | GCTGATGAACTCCTCAGGGA | |
|  | | |  |
| TP53BP1 | Forward Primer | CTCCAGACGCACAAAGAAAATCC | |
|  | Reverse Primer | ACCTGACTGATGGAACCACAT | |

**Appendix Table S7.** List of antibodies used.

| Western Blot | | | | |
| --- | --- | --- | --- | --- |
|  | **Vendor** | **Host** | **Clone** | **Cat No** |
| KMT2C | Santa Cruz | Rabbit | C-16 | 130173 |
| RBBP5 | Cell Signaling | Rabbit | D316P | 13171S |
| β−actin | Cell Signaling | Rabbit | 13E5 | 4970 |
| ATM | Cell Signaling | Rabbit | D2E2 | 2873 |
| ATR | Santa Cruz | Mouse | C-1 | sc-515173 |
| BRCA1 | Santa Cruz | Mouse | D-9 | sc-6954 |
| CHEK2 | Santa Cruz | Mouse | A-12 | Sc-5278 |
| TP53BP1 | Santa Cruz | Rabbit | H-300 | sc-22760 |
| RAD51 | Santa Cruz | Rabbit | H-92 | sc-8349 |
| Immunofluorescence | | | | |
|  | **Vendor** | **Host** | **Clone** | **Cat No** |
| γH2AX | Millipore | Mouse | JBW301 | 05-636 |
| RAD51 | Santa Cruz | Rabbit | H-92 | sc-8349 |
| TP53BP1 | Abcam | Rabbit |  | ab21083 |
| KMT2C | Abcam | Rabbit | acetyl-k2809 | ab40973 |
| α-tubulin-alexa488 | Cell Signaling | Mouse | DM1A | 8058 |
| Pankeratin | Dako | Mouse | AE1/AE3 | IR053 |
| Immunohistochemistry | | | | |
|  | **Vendor** | **Host** | **Clone** | **Cat No** |
| Ki67 | Abcam | Rabbit |  | ab15580 |
| Active Casp3 | Abcam | Rabbit |  | ab2302 |
| γH2AX | Millipore | Mouse | JBW301 | 05-636 |
| RAD51 | Santa Cruz | Rabbit | H-92 | sc-8349 |
| Chromatin IP | | | | |
|  | **Vendor** | **Host** | **Clone** | **Cat No** |
| H3 | Cell Signaling | Rabbit | D2B12 | L1620 |
| H3K4me3 | Cell Signaling | Rabbit |  | 9727 |
| H3K9ac | Cell Signaling | Rabbit | C5B11 | 9649P |
| H3K27ac | Cell Signaling | Rabbit | D5E4 | 8173 |
| Anti-Flag | Sigma | Mouse | M2 | F3165 |

**Appendix Figure S1. KMT2C expression in non-epithelial cancers.**

Expression levels (transcripts per million) of *KMT2C* in non-epithelial cancers. DLBCL: diffuse large B-cell lymphoma; LAML: acute myeloid leukemia; GBM: glioblastoma multiforme; LGG: brain lower grade glioma; SARC: sarcoma. The middle lines inside the boxes indicate the median (50th percentile). The lower and the upper box boundaries represent the 25th percentile and the 75th percentile, respectively. The lower and upper whiskers extend to the lowest and highest value, respectively, within the 1.5× interquartile range (box height) from the box boundaries.

**Appendix Figure S2. Cell proliferation and apoptosis in HTB9 (A) and T24 (B) shRNA control (Scr) and HTB9/KD1 cells.**

A, B Cell cycle analysis (left) and Annexin V staining (middle) indicates that proliferation and apoptosis levels are similar between control Scr and KMT2C/KD1 cells. Also, MTT colorimetric assays (right) support these observations. Values in the figure are mean ±SEM, n=3, student’s t-test was used.

**Appendix Figure S3. KMT2C binds onto the promoter of DDR and DNA repair genes.**

Bedgraphs indicating histone modifications and KMT2C binding (two different experiments) onto promoter regions of the indicated genes. Deposited ChIP-seq data (GSE97326) were analyzed with the IGV viewer. Sample number: H3K4me3: GSM2561639; H3K27ac: GSM2561637; KMT2C.1: GSM2561687; and KMT2C.2: GSM2561688.

**Appendix Figure S4. KMT2C controls the expression of DDR and DNA repair genes in various cancers.**

Boxplots indicating a statistically significant correlation in expression levels between *KMT2C* and indicated genes in Glioblastoma multiforme (GBM, n=58), Low Grade Glioma (LGG, n=172), Acute Myeloid Leukemia (AML, n=58), Diffuse Large B-cell Lymphoma (DLBL, n=16), Sarcoma (SARC, n=80) and Breast Invasive Carcinoma (BRIC, n=320). RNA-Seq data were obtained from the TCGA through cbioportal.org. Mann-Whitney U test was used. * designates *P* value<0.05, ** *P* value<0.01, *** *P* value<0.001 and **** *P* value<0.0001.

**Appendix Figure S5. KMT2C expression is independent of TP53 mutation and expression status in human epithelial cancers.**

(A) Boxplots indicating *KMT2C* expression (Y axis) and *TP53* mutation status (X axis) in the indicated human tumors. A positive correlation (p=0.022) is observed only in the case of BC. Mann-Whitney U test was used.

(B) Table indicating the degree of co-occurrence between *KMT2C* and *TP53* mutation in the human epithelial tumors. Only in the case of lung adenocarcinoma (LUAD) did we observe a statistically significant (p<0.001) co-occurrence. For (A) and (B) data were obtained from the TCGA through cbioportal.org. Fisher's exact test is used by the platform.

(C) Boxplots indicating *KMT2C* expression (Y axis) and *TP53* mutation status (X axis) in human cell lines. Only in the case of NSCLC did we observe a statistically significant negative correlation (p<0.0001) between *TP53* mutations and *KMT2C* expression. Data were obtained directly from the Broad Institute server. Mann-Whitney U test was used.

For A and C boxplots, the middle lines inside the boxes indicate the median (50th percentile). The lower and the upper box boundaries represent the 25th percentile and the 75th percentile, respectively. The lower and upper whiskers extend to the lowest and highest value, respectively, within the 1.5× interquartile range (box height) from the box boundaries.
