# Supplementary material for: The lysine‐specific methyltransferase KMT2C/MLL3 regulates DNA repair components in cancer
Source: EMBO Rep. 2019 Jan 21;20(3):e46821. doi: 10.15252/embr.201846821 (PMC6399616; doi:10.15252/embr.201846821)
Supplement: Supplementary file 2 — Expanded View Figures PDF [file EMBR-20-e46821-s002.pdf]

Expanded View Figures

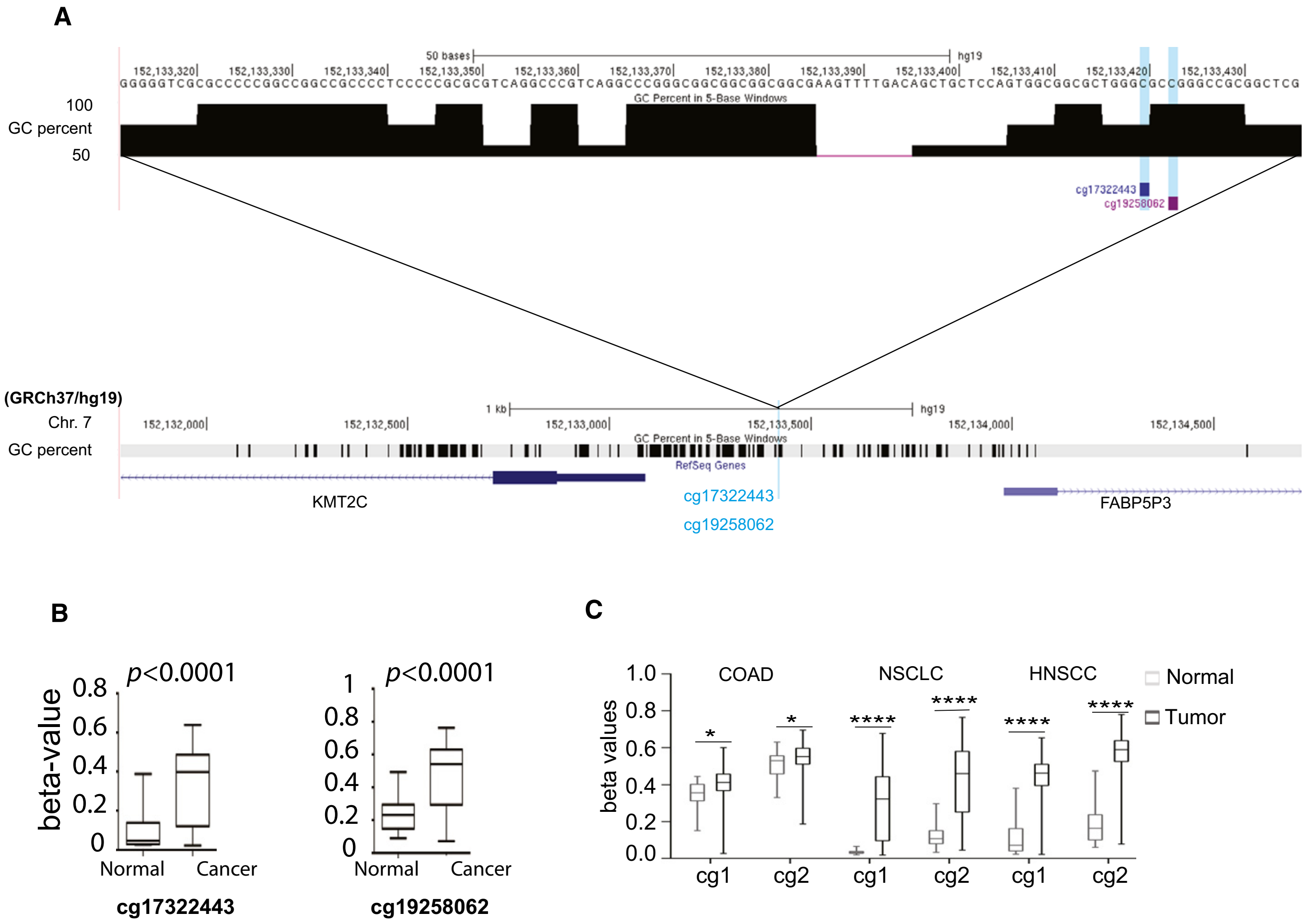

Figure EV1. KMT2C promoter methylation in human cancers.

A Schematic of the upstream promoter region of the *KMT2C* locus indicating the position and sequence of methylation detection probes within the CpG island (located at chr7:152435133–152437025, assembly GRCh38/hg38) that encompasses the *KMT2C* promoter region.

B Comparison of the methylation levels of the above probes in tumor samples and normal bladder tissue. Methylation data were obtained from TCGA through the MethHC database for $n = 21$ healthy/tumor pairs. Wilcoxon matched-pairs signed-rank test was used.

C Tumor vs. normal paired comparison of the methylation levels in the *KMT2C* promoter in various cancer types; cg1: cg17322443; cg2: cg19258062. Methylation data were obtained from the MethHC database (Huang *et al* [29]). BC: $n = 21$, COAD: $n = 21$, NSCLC: $n = 70$, HNSCC: $n = 50$. For NSCLC analysis, separate cohorts from adenocarcinoma and squamous cell carcinoma were combined. Separate analysis of the two NSCLC subtypes yielded the same results. Wilcoxon matched-pairs signed-rank test was used. * designates *P*-value < 0.05 and ****P-value < 0.0001.

© 2019 The Authors

Figure EV2. Cells lacking KMT2C are HR-deficient.

A Immunofluorescence of γH2AX foci and quantitation in control (Scr) and *KMT2C*-knockdown (KMT2C/KD1 and KD2) T24 cells. *BRCA1* knockdown (BRCA1/KD) is used as control. Scale bars indicate 10 μm. Values in the plot correspond to mean ± SEM. Data from three experiments were analyzed with Student's *t*-test. * designates *P*-value < 0.05, and ** designates *P*-value < 0.01. Remaining protein levels of BRCA1 are also shown for both HTB9 (referring to Fig 5A) and T24 are also shown.

B Frequency of RAD51 foci in cisplatin-treated T24 control (Scr) and *KMT2C*-knockdown (KD1) cells. Scale bars indicate 10 μm. Values in the plot correspond to mean ± SEM. Data from three experiments were analyzed with Student's *t*-test. * designates *P*-value < 0.05, and ** designates *P*-value < 0.01.

C Sister chromatid exchange assay with cisplatin-treated T24 control (Scr) and *KMT2C*-knockdown (KD1) cells. Results were obtained from 15 metaphases per group. White arrowheads indicate sister chromatid exchange events.

D DNA fiber assay on control (Scr) and *KMT2C*-knockdown (KD1) T24 cells. *BRCA1*-knockdown cells are used as controls. Experiments performed with or without hydroxyurea (HU) treatment under the conditions indicated in Fig 5D. The length of minimum 100 fibers from each condition was measured. Red horizontal lines indicate the median tract length in each group.

Source data are available online for this figure.

 © 2019 The Authors

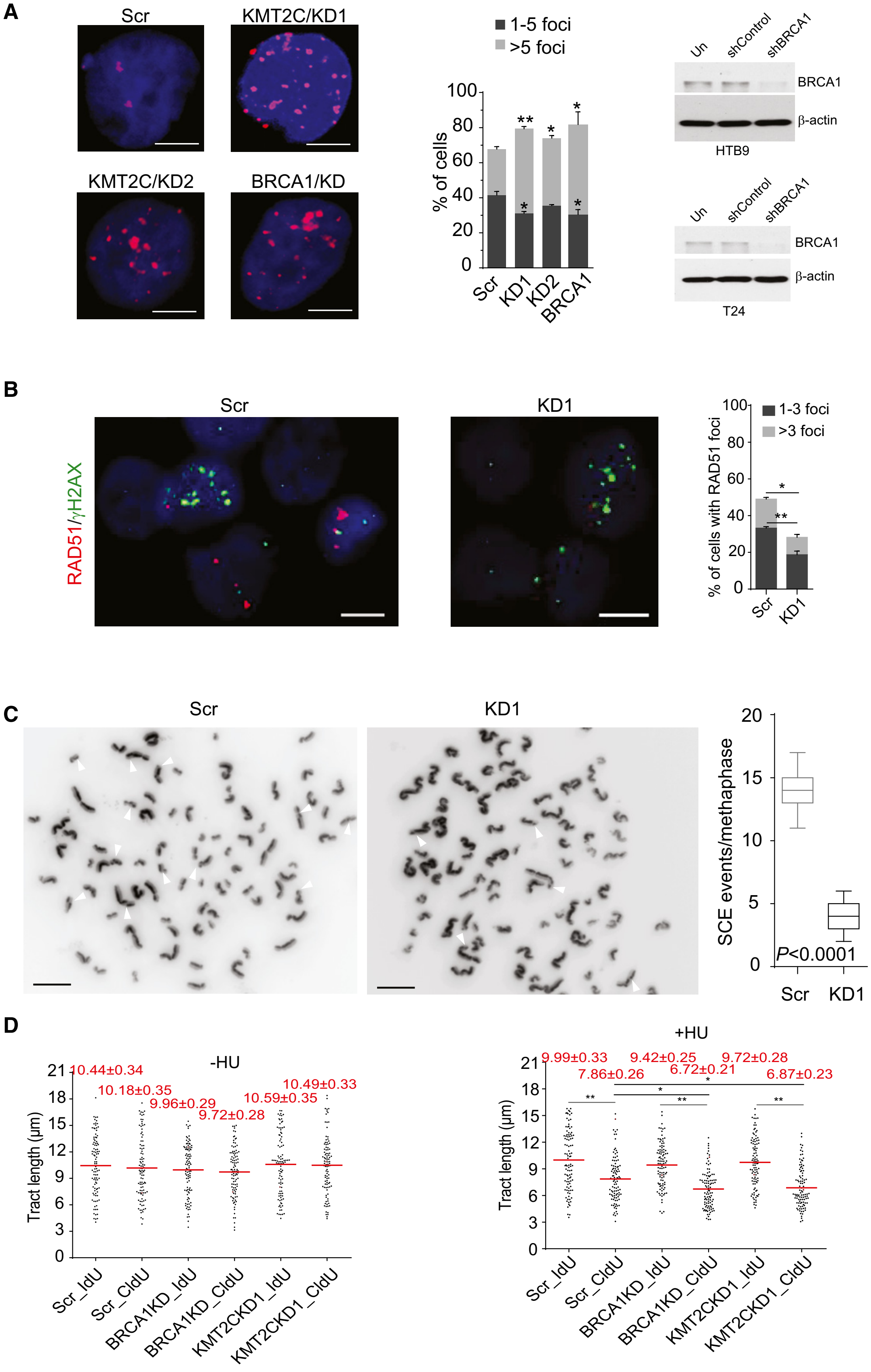
A
Scr
KMT2C/KD1
KMT2C/KD2
BRCA1/KD
1-5 foci
>5 foci
% of cells
Scr
KD1
KD2
BRCA1
Un
shControl
shBRCA1
BRCA1
β-actin
HTB9
T24
B
Scr
KD1
RAD51/γH2AX
1-3 foci
>3 foci
% of cells with RAD51 foci
C
Scr
KD1
SCE events/methaphase
P<0.0001
D
-HU
+HU
Tract length (µm)
10.44±0.34
10.18±0.35
9.96±0.29
9.72±0.28
10.59±0.35
10.49±0.33
9.99±0.33
7.86±0.26
9.42±0.25
6.72±0.21
9.72±0.28
6.87±0.23
Scr_IdU
Scr_CldU
BRCA1KD_IdU
BRCA1KD_CldU
KMT2CKD1_IdU
KMT2CKD1_CldU

Figure EV2.

Figure EV3. KMT2C loss leads to PARP1/2 dependence for DNA repair.

A Frequency of TP53BP1 foci in cisplatin-treated T24 control (Scr) and *KMT2C*-knockdown (KD1) cells. Size bars in microscopy panels correspond to 10 μm. In the plot, bars represent mean ± SEM from n = 3 experiments.

B Frequency of chromosome fusions obtained from IR-treated (schematic) T24 control (Scr) and *KMT2C*-knockdown (KD1) cells. Representative karyotypes are shown. Size bars in karyotype panels correspond to 5 μm. White arrows indicate chromosome fusions. In the plot, bars represent mean ± SEM from n = 3 experiments.

C Frequency of chromosome fusions in IR-treated T24 control (Scr) and *KMT2C*-knockdown (KD1) cells upon treatment with SCR7 and olaparib. Bars represent mean ± SEM from n = 3 experiments. Throughout the figure, Mann–Whitney *U*-test was used.

© 2019 The Authors

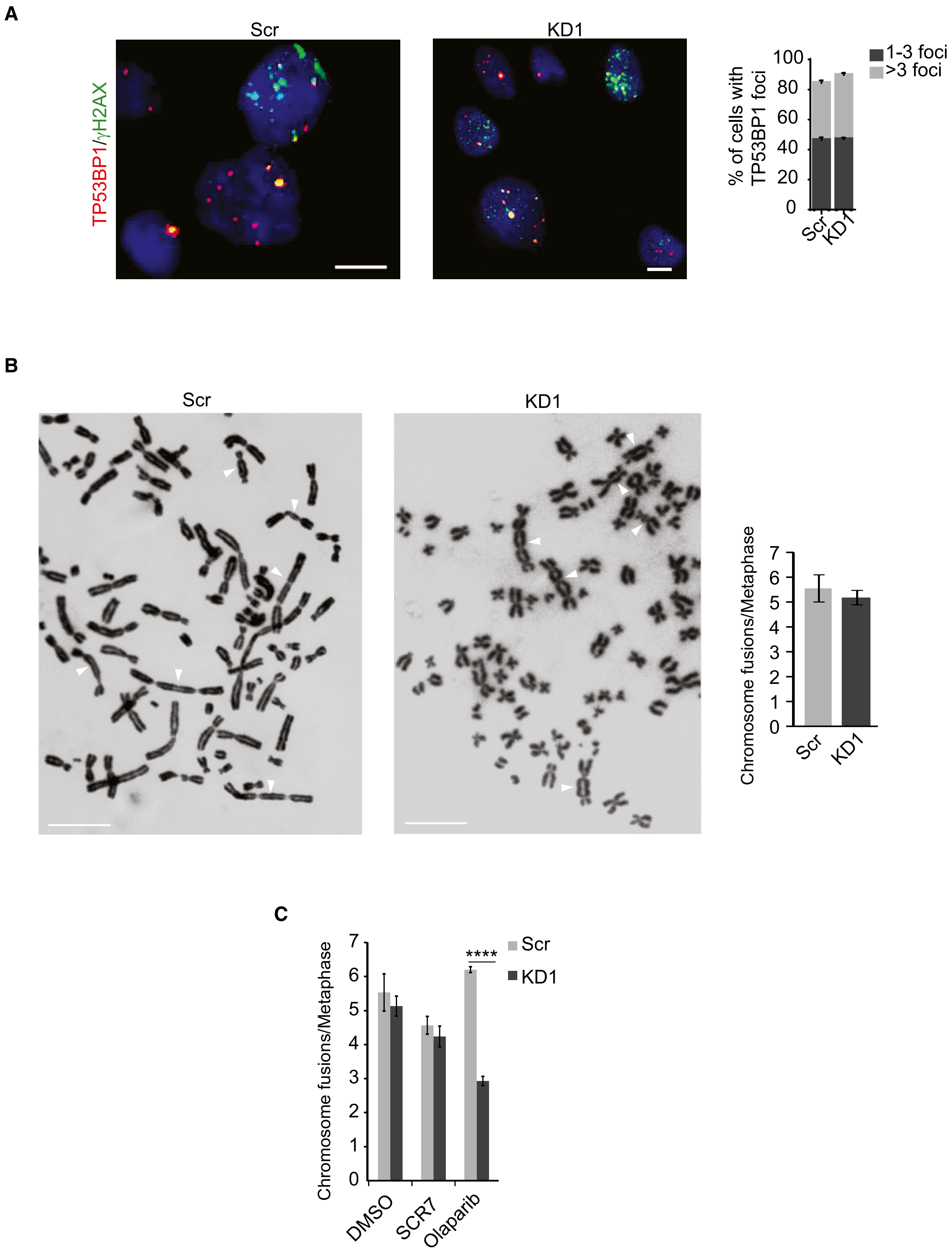
A
Scr
KD1
TP53BP1/γH2AX
% of cells with TP53BP1 foci
100
80
60
40
20
0
Scr
KD1
1-3 foci
>3 foci
B
Scr
KD1
Chromosome fusions/Metaphase
7
6
5
4
3
2
1
0
Scr
KD1
C
Chromosome fusions/Metaphase
7
6
5
4
3
2
1
0
DMSO
SCR7
Olaparib
****
Scr
KD1

Figure EV3.

Figure EV4. KMT2C loss leads to PARP1/2 dependence for DNA repair.

A, B Frequency of radial structures in IR-treated HTB9/KD1 (A) and T24/KD1 (B) cells upon ligase III and ligase IV knockdown (top left), and Western blot analysis indicating respective leftover protein levels (top right). Representative karyotypes are shown. Values in the plot indicate mean ± SEM. Analysis of three experiments was performed using Student's *t*-test. * designates *P*-value < 0.05 and ****P*-value < 0.001.

Source data are available online for this figure.

© 2019 The Authors

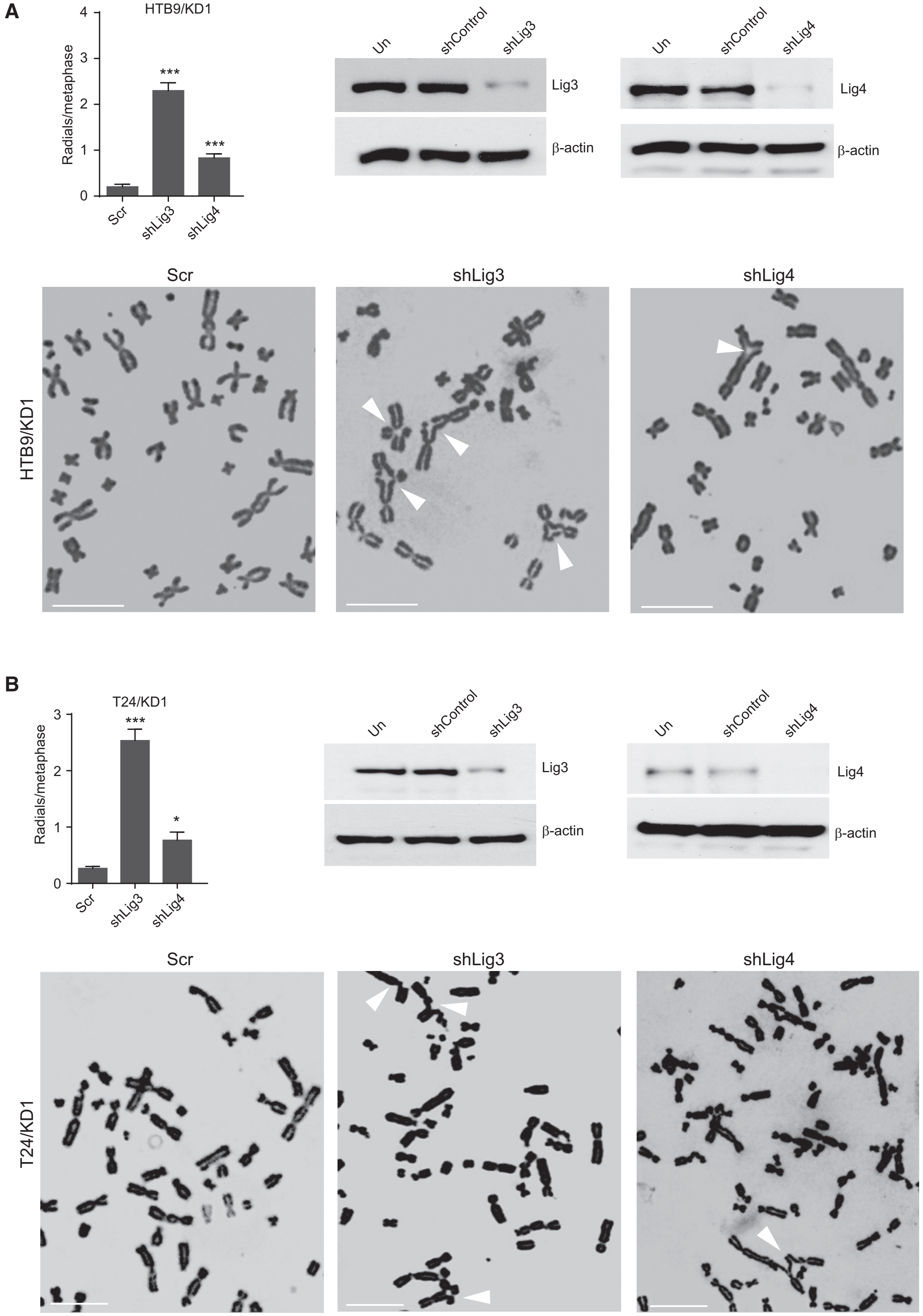
A
HTB9/KD1
Radials/metaphase
4
3
2
1
0
***
***
Scr
shLig3
shLig4
Un
shControl
shLig3
Lig3
β-actin
Un
shControl
shLig4
Lig4
β-actin
Scr
shLig3
shLig4
HTB9/KD1
B
T24/KD1
Radials/metaphase
3
2
1
0
***
*
Scr
shLig3
shLig4
Un
shControl
shLig3
Lig3
β-actin
Un
shControl
shLig4
Lig4
β-actin
Scr
shLig3
shLig4
T24/KD1

Figure EV4.

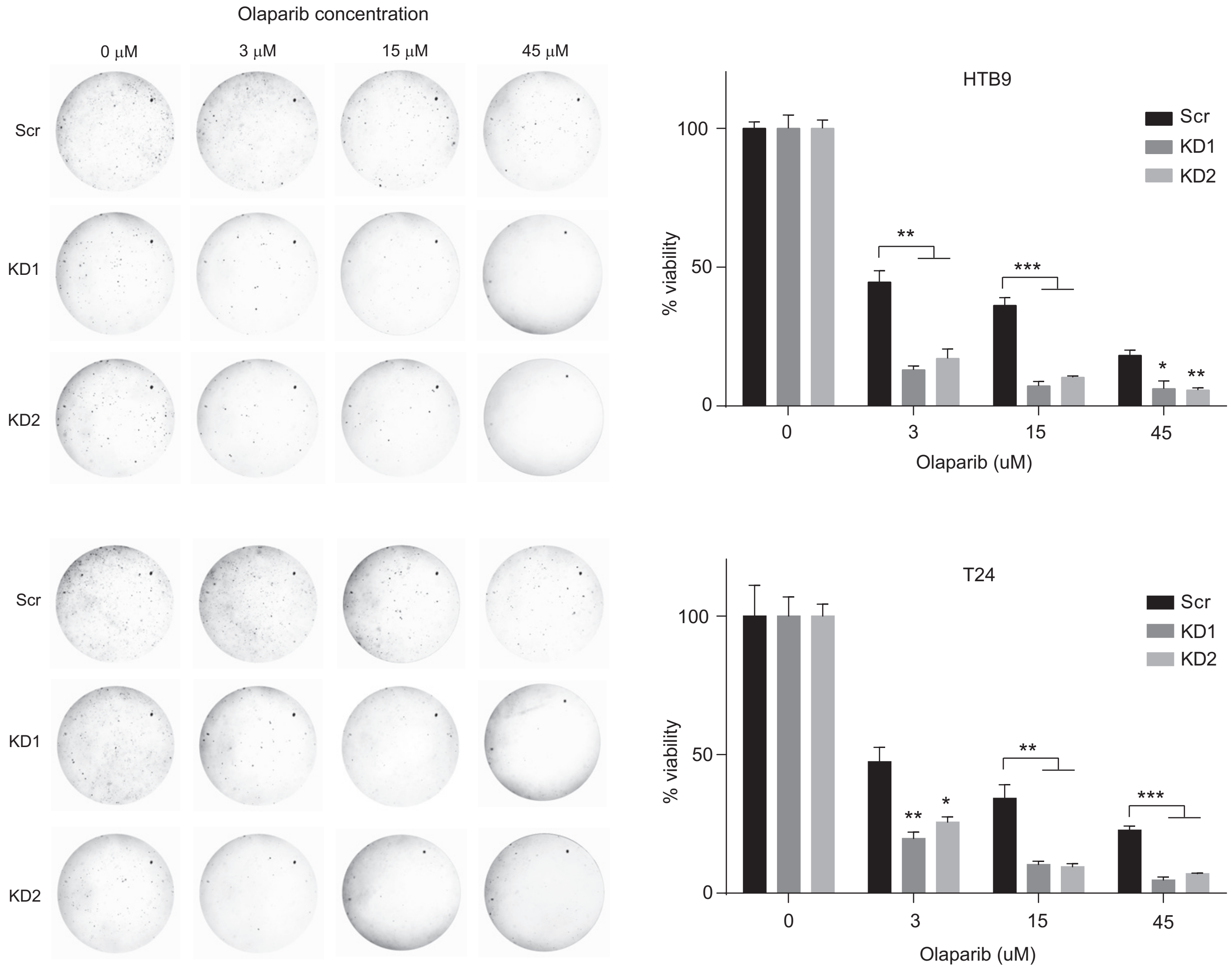

Figure EV5. *In vitro* clonogenic assays indicating PARPi sensitivity of KMT2C/KD cells.

Representative photographs (left) and number of colonies (*y*-axis) generated by HTB9 and T24 (Scr and KMT2C/KD1) cells treated with increasing concentrations of olaparib (*x*-axis). Cells were seeded and grown for 20 days at which point the experiment was concluded and dishes were photographed. Values in the plot indicate mean number of colonies ± SEM. Analysis of three experiments was performed using Student's *t*-test. * designates *P*-value < 0.05, ** designates *P*-value < 0.01, and *** designates *P*-value < 0.001.

© 2019 The Authors
