# Supplementary material for: The lysine‐specific methyltransferase KMT2C/MLL3 regulates DNA repair components in cancer
Source: EMBO Rep. 2019 Jan 21;20(3):e46821. doi: 10.15252/embr.201846821 (PMC6399616; doi:10.15252/embr.201846821)
Supplement: Supplementary file 3 — Table EV1 [file EMBR-20-e46821-s003.docx]

**Table EV1.** KMT2C mutations and predicted activity

| **Patient** | **Grade^a^** | **Stage** | **Nucleotide^b^** | **Protein Domain** | **Amino acid^c^** | **PolyPhen2^d^** |
| --- | --- | --- | --- | --- | --- | --- |
| TCC1 | High | T2 | c.C919T | PHD1 | H307Y | D/D |
| TCC5 | High | T3 | c.G1345T | PHD2 | Q448H | B/B |
| TCC16 | High | T3 | c.T1061A | PHD1 | L354I | D/D |
| TCC24 | High | T1 | c.G1193A | PHD1 | G398R | D/D |
| TCC32 | High | T1 | c.A1257T | PHD2 | Q419H | D/D |
| TCC33 | Low | Ta | c.G764T | PHD1 | C255F | D/D |
| TCC35 | High | T2 | c.G839T | PHD1 | G280V | D/D |
| TCC43 | High | T2 | c.A1365G | PHD2 | I455M | B/B |
| TCC48 | High | T3 | c.14164del112nt | PHD1 | F4772fs5X |  |
| TCC53 | High | T3 | c.853del | PHD1 | R334fs54X |  |
| TCC67 | High | T4 | c.G839T | PHD1 | C255F | D/D |
| TCC85 | Low | Ta | c.C485T | AT | P162L | D/D |
| TCC90 | Low | T1 | c.T14058A | SET2 | N4686K | D/D |
|  |  |  | c.A1257T | PHD2 | Q419H | D/D |
| TCC99 | High | T3 | c.T14029C | SET2 | p.S4677T | D/D |
|  |  |  | c.C13873T | SET2 | L4625I | D/D |
| TCC116 | Low | Ta | c.G1345T | PHD2 | Q448H | B/B |
| TCC142 | High | T1 | c.A1393T | PHD2 | N465Y | D/D |

^a^ WHO 2004

^b^ Ref: XM_005250025.3

^c^ Ref: XP_005250082.1

^d^ D: damaging; B: benign
