# Supplementary material for: The lysine‐specific methyltransferase KMT2C/MLL3 regulates DNA repair components in cancer
Source: EMBO Rep. 2019 Jan 21;20(3):e46821. doi: 10.15252/embr.201846821 (PMC6399616; doi:10.15252/embr.201846821)
Supplement: Supplementary file 4 — Table EV2 [file EMBR-20-e46821-s004.docx]

| TISSUE | Cell line | Clone | KMT2C expression over Scr |
| --- | --- | --- | --- |
| BLADDER | HTB9 | Scr |  |
|  |  | KD1 | 0.20±0.05 |
|  |  | KD2 | 0.31±0.07 |
|  | T24 | Scr |  |
|  |  | KD1 | 0.22±0.02 |
|  |  | KD2 | 0.17±0.02 |
|  | RT4 | Scr |  |
|  |  | KD1 | 0.30±0.02 |
|  | TCCSUP | SCR |  |
|  |  | KD1 | 0.61±0.03 |
| COLORECTAL | DLD1 | Scr |  |
|  |  | KD1 | 0.47±0.02 |
|  | CACO-2 | Scr |  |
|  |  | KD1 | 0.68±0.02 |
|  | T84 | Scr |  |
|  |  | KD1 | 0.45±0.02 |
| HEAD & NECK | BB49 | Scr |  |
|  |  | KD1 | 0.53±0.04 |
|  | Cal-33 | Scr |  |
|  |  | KD1 | 0.50±0.05 |
|  | SCC090 | Scr |  |
|  |  | KD1 | 0.73±0.12 |
| LUNG | H1437 | Scr |  |
|  |  | KD1 | 0.24±0.02 |
|  | H1792 | Scr |  |
|  |  | KD1 | 0.75±0.09 |
|  | A549 | Scr |  |
|  |  | KD1 | 0.59±0.05 |

**Table EV2.** KMT2C expression after knockdown.
