# Supplementary figures and images for: The lysine‐specific methyltransferase KMT2C/MLL3 regulates DNA repair components in cancer
Source: EMBO Rep. 2019 Jan 21;20(3):e46821. doi: 10.15252/embr.201846821 (PMC6399616; doi:10.15252/embr.201846821)

Figure EV4

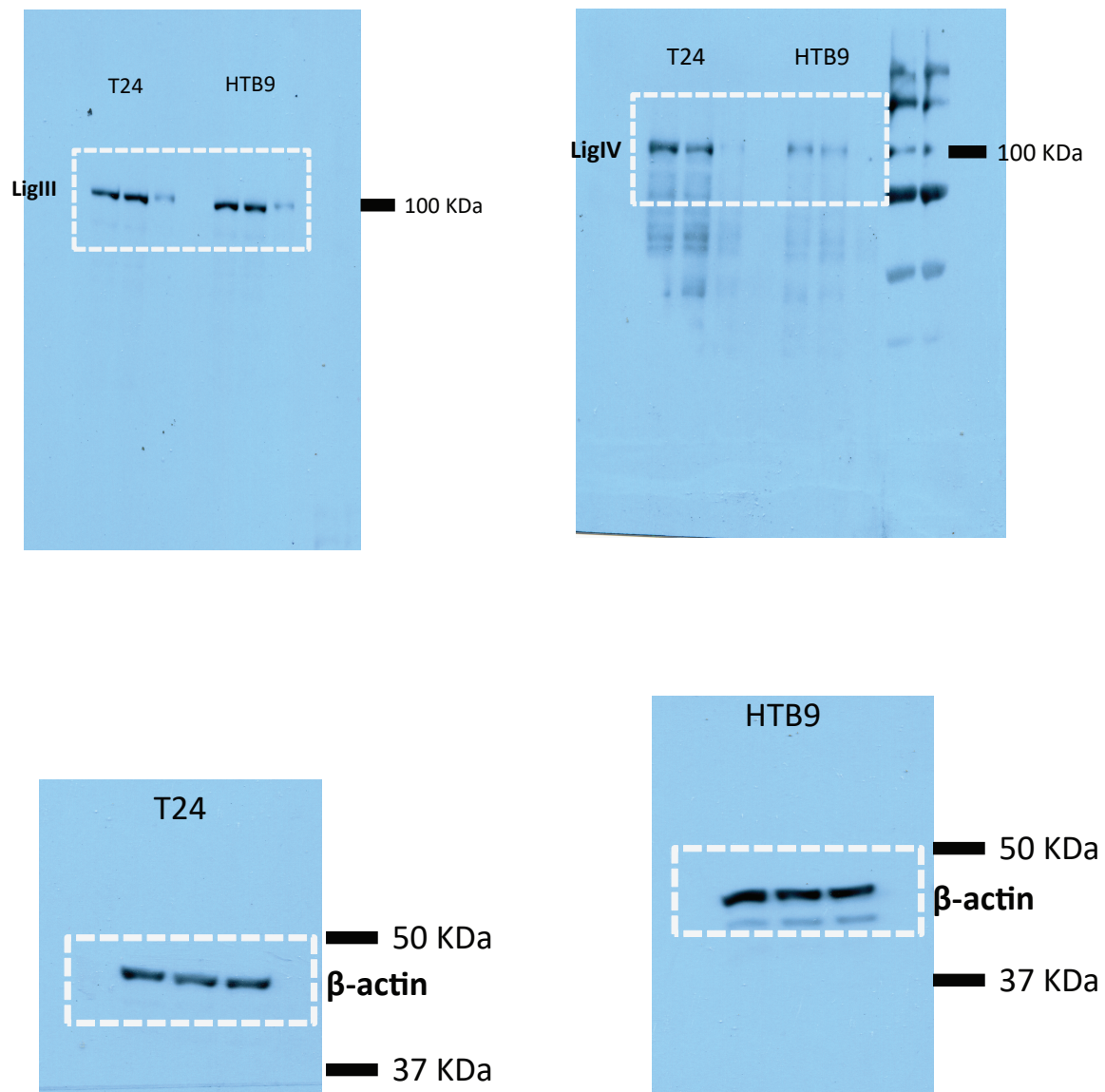

Supplement: Supplementary file 5 — Source Data for Expanded View [file EMBR-20-e46821-s008.zip › embr201846821-sup-0009-SDataFigEV4.pdf]

Figure EV2

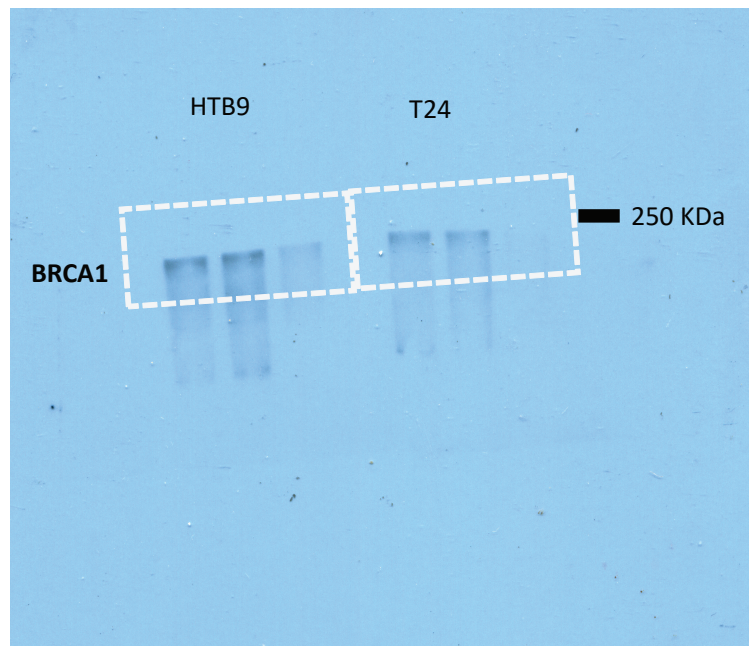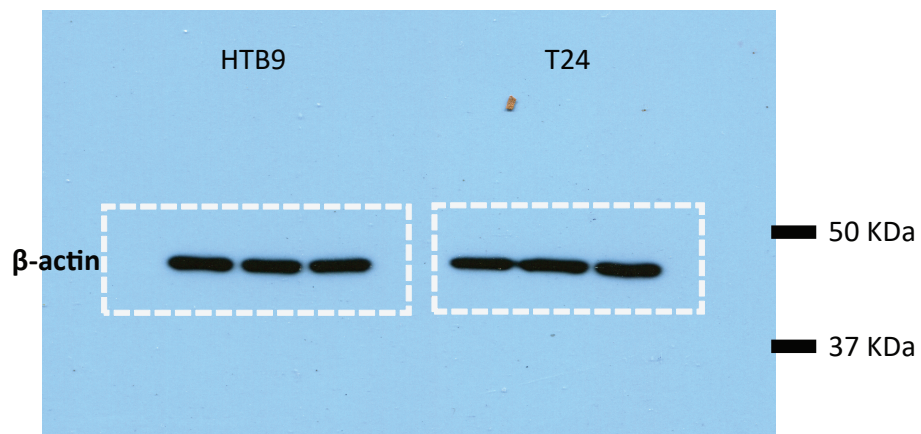

Supplement: Supplementary file 5 — Source Data for Expanded View [file EMBR-20-e46821-s008.zip › embr201846821-sup-0008-SDataFigEV2.pdf]

Figure 1

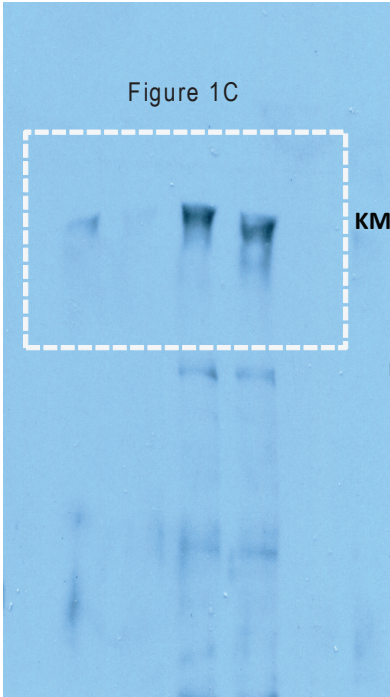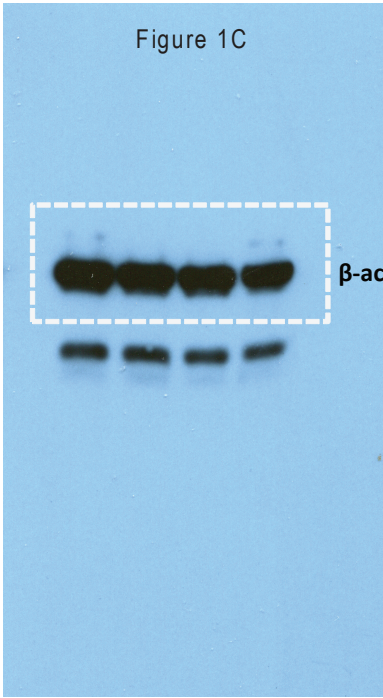

Supplement: Supplementary file 7 — Source Data for Figure 1 [file EMBR-20-e46821-s005.pdf]

Figure 2

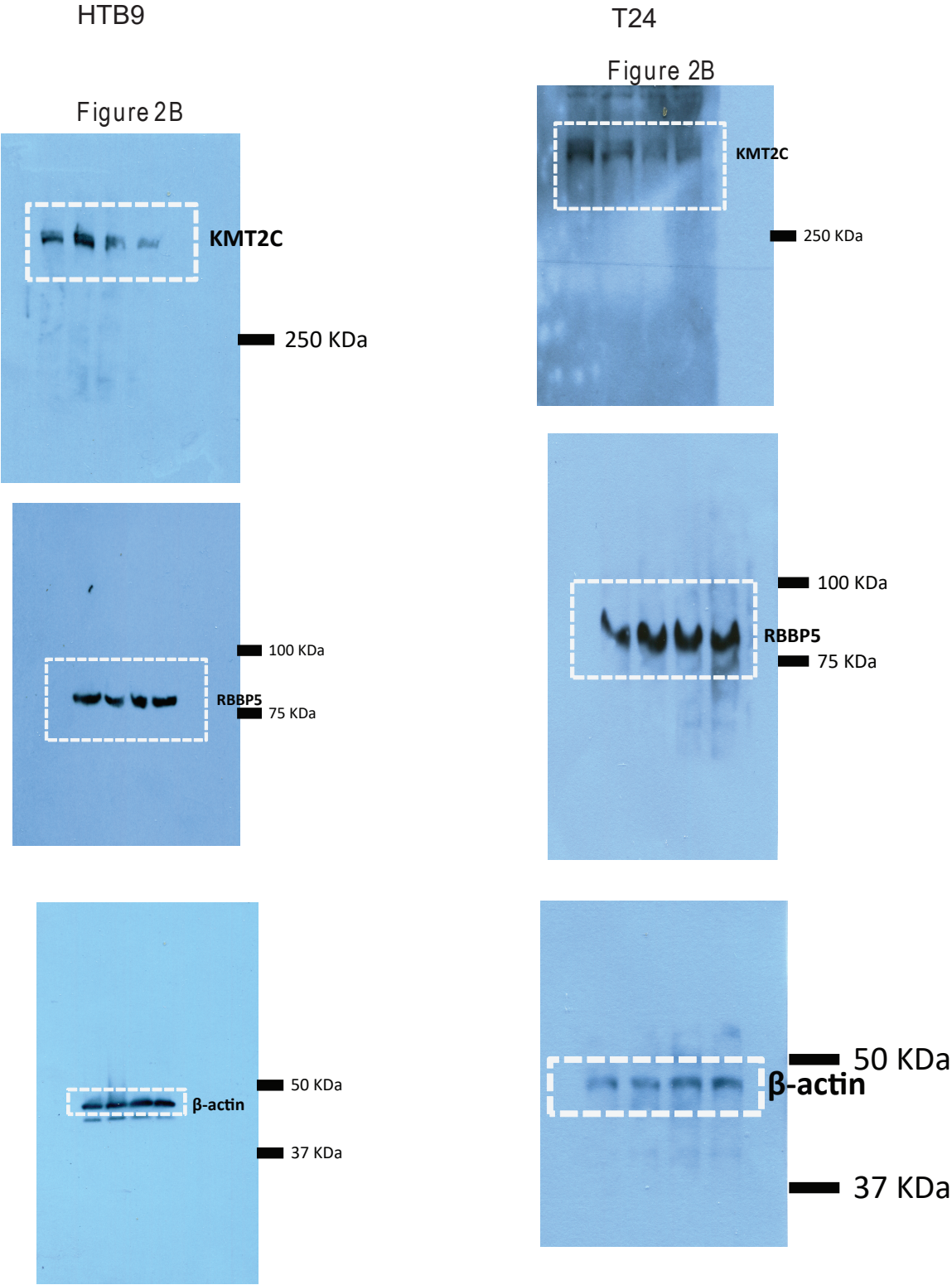

Supplement: Supplementary file 8 — Source Data for Figure 2 [file EMBR-20-e46821-s006.pdf]

Figure 3

Figure 3E

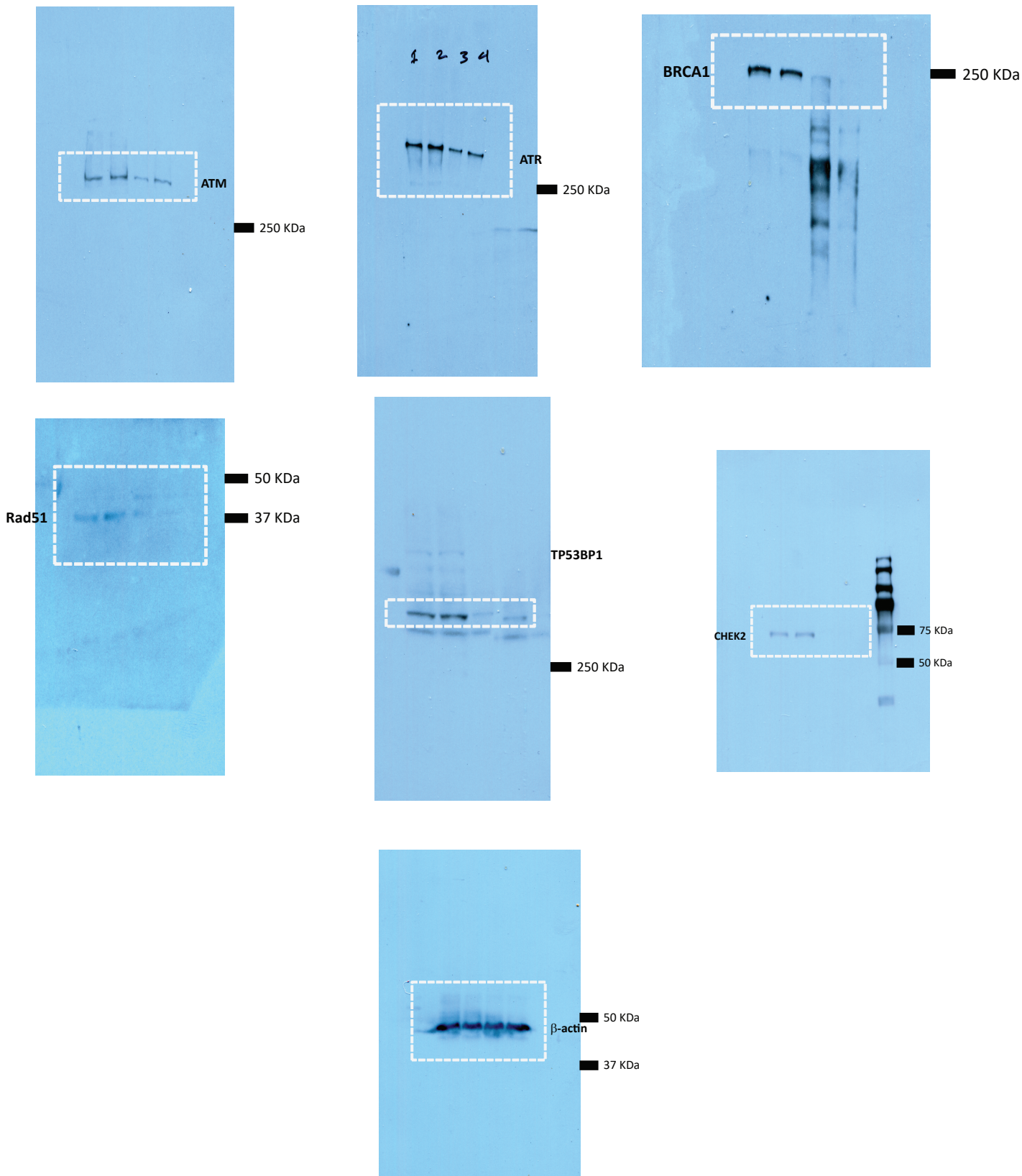

Supplement: Supplementary file 9 — Source Data for Figure 3 [file EMBR-20-e46821-s007.pdf]
